# Self-assembly–based posttranslational protein oscillators

**DOI:** 10.1126/sciadv.abc1939

**Published:** 2020-12-16

**Authors:** Ofer Kimchi, Carl P. Goodrich, Alexis Courbet, Agnese I. Curatolo, Nicholas B. Woodall, David Baker, Michael P. Brenner

**Affiliations:** 1 Harvard University School of Engineering and Applied Sciences, Cambridge, MA 02138, USA.; 2 Department of Biochemistry, University of Washington, Seattle, WA 98105, USA.; 3 Institute for Protein Design, University of Washington, Seattle, WA 98105, USA.; 4 Howard Hughes Medical Institute, University of Washington, Seattle, WA 98105, USA.; 5 Kavli Institute for Bionano Science and Technology Harvard University, Cambridge, MA 02138, USA.

## Abstract

Recent advances in synthetic posttranslational protein circuits are substantially impacting the landscape of cellular engineering and offer several advantages compared to traditional gene circuits. However, engineering dynamic phenomena such as oscillations in protein-level circuits remains an outstanding challenge. Few examples of biological posttranslational oscillators are known, necessitating theoretical progress to determine realizable oscillators. We construct mathematical models for two posttranslational oscillators, using few components that interact only through reversible binding and phosphorylation/dephosphorylation reactions. Our designed oscillators rely on the self-assembly of two protein species into multimeric functional enzymes that respectively inhibit and enhance this self-assembly. We limit our analysis to within experimental constraints, finding (i) significant portions of the restricted parameter space yielding oscillations and (ii) that oscillation periods can be tuned by several orders of magnitude using recent advances in computational protein design. Our work paves the way for the rational design and realization of protein-based dynamic systems.

## Teaser

Mathematical models for two experimentally realizable posttranslational protein oscillators are constructed.

## INTRODUCTION

Protein oscillators play a major regulatory role in organisms ranging from prokaryotes to humans. In most known biological cases, the oscillation is realized through transcription/translation cycles. Few examples of purely posttranslational oscillators have been found in biology (*1*, *2*). At the same time, posttranslational protein circuits are increasingly sought after for synthetic applications, since they have the potential to exhibit faster response to environment changes, allow for more direct control over the circuit behavior, be directly coupled to a functional output, and can be used in contexts that do not include the vast genetic apparatus (*3*–*5*). While significant recent work has enabled the design of synthetic posttranslational protein–based logic gates (*4*, *5*), engineering tunable dynamic phenomena such as oscillations in a synthetic posttranslational context remains an outstanding challenge (*6*, *7*).

The best-studied example of biological posttranslational protein oscillators is the KaiABC system in cyanobacteria (*8*). By placing only the proteins KaiA, KaiB, and KaiC in a test tube, along with abundant adenosine triphosphate, the KaiC proteins collectively get sequentially phosphorylated and dephosphorylated, forming an oscillatory cycle (*9*, *10*). While the KaiC proteins generally exist in a hexameric state, monomers are shuffled among the hexamers during only a certain phase of the oscillatory cycle (*11*). The KaiABC system demonstrates that protein oscillators need not use transcription/translation cycles or large numbers of components to achieve oscillatory behavior.

Motivated by the KaiABC system, we set out to design a protein-based oscillator that could be reconstituted in vitro using only a small number of components at relatively high copy numbers, so that any resulting oscillations are not stochastic. To facilitate the future translation of this theoretical study to an experimental system, we base the architecture of our system on biochemical constraints and on a design space navigable through computational protein design. We constrain the kinetic reaction network to only include three protein species and to only allow reversible binding and phosphorylation/dephosphorylation enzyme reactions.

As in KaiABC, the oscillating system will cycle through periods of a protein species being phosphorylated or not. When the target protein is phosphorylated, the phosphorylation must induce a change to propel the global state along the oscillatory cycle. This change can be achieved by affecting the enzyme kinetics of the kinase and phosphatase in one of two ways: either by altering the conformation of the target protein or by directly modifying the kinase and phosphatase enzymes. We first consider systems that do not modify the kinase or phosphatase.

The simplest such system, a protein with one phosphorylation site being modified by a kinase and a phosphatase, cannot yield oscillations regardless of parameter choices (*7*). When two phosphorylation sites are included, oscillations are possible only under the assumption that each of the four possible phosphorylation states has significantly different rates of subsequent phosphorylations and dephosphorylations (*7*). While biology seems to have designed a system in KaiABC capable of undergoing the many conformational changes necessary to implement this form of oscillations (*9*), the design of even two (let alone several) protein structures from the same sequence remains a significant challenge for the field of computational protein design (*12*).

These challenges are not unique to molecules with two phosphorylation sites. For example, since oscillations for molecules with two phosphorylation sites are effected by enzyme sequestration (*7*), we consider a molecule containing a single phosphorylation site alongside a kinase- or phosphatase-sequestering domain (or a binding domain for an external compound that itself contains an enzyme-binding domain). These systems are capable of producing oscillations—but only if phosphorylation and binding accompany a significant conformational change in the molecule that modifies the rate constants of subsequent reactions. Even assuming that such a conformational change were designed, we have found no evidence of sustained oscillations in these systems within the parameter regimes of typical binding/unbinding rate constants and typical kinase and phosphatase activity (i.e., the catalytic rate and Michaelis constants *k*_cat_ and *K*_M_, discussed further below). See section S1 for further discussion.

Systems that focus on modifications to the enzymes themselves are therefore more likely candidates for the production of experimentally realizable oscillations. Biology has found several ways to tie phosphorylation to enzymatic activity. The most straightforward conceptually, having the activity of an enzyme dependent on its own phosphorylation state (*13*) remains a challenge to implement in the context of computational protein design (*14*). However, the field has achieved remarkable success in the design of protein-protein interactions (*15*) which can be modified by phosphorylation (*16*, *17*).

Bootstrapping off of this success, we consider proteins that self-assemble into multimeric functional kinases and phosphatases (*18*, *19*). We are motivated, in part, by the success of using split proteases to implement posttranslational protein–based logic gates (*4*, *5*). In our design, when the proteins’ binding interfaces are phosphorylated, their self-assembly is impeded, reducing the concentration of functional enzymes available in the system.

**Fig. 1:**
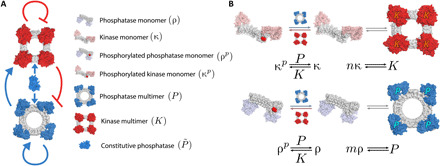
Bounded self-assembly oscillator. (**A**) Oscillator topology. By phophorylating monomers, kinase multimers (red; top) inhibit their own and phosphatase multimer (blue; bottom) self-assembly. Similarly, phophatase multimers counteract this inhibition, as do constitutive phosphatases (center). (**B**) Bounded self-assembly reactions. Monomers contain two halves of a split enzyme: either kinase (red; top) or phosphatase (blue; bottom). Monomers can self-assemble into multimers of specified size (here, tetramers are pictured, corresponding to *n* = *m* = 4). Kinase (phosphatase) multimers can (de)phosphorylate the monomers. A constitutive phosphatase is also able to dephosphorylate the monomers (not pictured). Phosphorylated monomers cannot participate in the self-assembly. Reactions are shown in pictorial form above each corresponding chemical equation. The full set of differential equations corresponding to these reactions is given in eq. S1.

Oscillations are thus achieved by the push and pull of two opposing factors: Self-assembled kinases inhibit the self-assembly of new proteins by phosphorylating both kinase and phosphatase monomers; meanwhile, self-assembled phosphatases counteract this inhibition. Incoherent inputs such as these are known to enhance the robustness of oscillations (*20*). Our overall oscillator design is motivated by analogy to known successful oscillators, particularly the dual-feedback genetic oscillator (*1*, *21*–*23*). Just as that oscillator relies on the interplay between the inhibiting effects of LacI and the activating effects of AraC, our oscillations rely on the interplay between kinase and phosphatase multimers, which respectively inhibit and activate their own self-assembly (Fig. 1A).

The rest of our manuscript is organized as follows. First, we describe the oscillatory circuits and their experimental constraints. Next, we develop simplified mathematical models for two distinct protein-based oscillators: In one, multimers are designed to form closed, bounded structures; in the other, they form unbounded fibers. We show that simple analytical formulae describing the first oscillator can predict both the regions of parameter space admitting oscillations and the oscillations’ resulting frequencies. We then demonstrate that the second oscillator design is complementary to the first in that it can admit robust and experimentally realizable oscillations in a regime of parameter space where the first cannot. Finally, we discuss the significance of our findings.

## RESULTS

### Self-assembly–based protein oscillators are designed within experimental constraints

#### Designed synthetic oscillators rely on few protein species with specified interactions

The main components of our oscillators are two proteins, which we call κ and ρ. Each individual protein of type κ (ρ) has two complementary parts of a split kinase (phosphatase) and a phosphorylation site. When the respective sites are dephosphorylated, copies of protein κ (ρ) can self-assemble into a functional kinase (phosphatase), which we call *K* (*P*). Thus, self-assembled kinases inhibit the self-assembly of new proteins, while self-assembled phosphatases counteract the inhibition. In addition to the proteins κ and ρ, we include a constitutive phosphatase $\tilde{P}$; without it, a fixed point where all proteins are phosphorylated can preclude oscillations (see section S2).

The resulting circuit topology (Fig. 1A) is analogous to that used in the dual-feedback genetic oscillator (*1*, *23*). The multimeric kinase plays an analogous role to the LacI protein in the genetic oscillator, repressing the production of new multimers; the multimeric phosphatase plays an analogous role to that of the AraC protein, activating the production (or more precisely, counteracting the kinase inhibition).

Two related networks based on these proteins can be designed. In the first, self-assembly is into closed symmetric homomultimers of specified size; in the second, the monomers are designed such that they self-assemble into one-dimensional unbounded fibers.

#### Experimental realizability constrains parameter sets

Because we are motivated by experimental feasibility, we consider only physically realizable parameters for our models. Binding rates *k*_b_ are typically in the range 10^−2^ to 10^0^ μM^−1^s^−1^ (*24*) with dissociation constants *k*_d_ typically in the 10^−3^ to 10^3^ μM range (*25*). Both of these quantities can be tuned on the basis of the geometry, energy, and symmetry of the binding interface between the proteins, which we assume here to be designed de novo. Less straightforward to design are the Michaelis constants and catalytic rates of the kinase and phosphatase, especially since these depend not only on the enzyme but on the substrate. Mutational screens can be used to adjust these parameters, but predicting the effect of a mutation on *k*_cat_ or *K*_M_ is highly nontrivial (*26*). We were unable to find studies measuring kinase and phosphatase rates on the same substrate. Instead, as a standard to demonstrate physical realizability, we consider the parameters for sample Ser/Thr enzymes: wild-type λ-phosphatase acting on para-nitrophenylphosphate (*k*_cat_ = 2.0 × 10^3^ s^−1^; *K*_M_ = 1.0 × 10^4^ μM) and wild-type MST4 (kinase) acting on the short peptide chain NKGYNTLRRKK (*k*_cat_ = 3.1 s^−1^; *K*_M_ = 14 μM) (*26*, *27*). We assume throughout that the constitutive phosphatase $\tilde{P}$ is governed by the same enzymatic rate constants as the self-assembled *P*. We also treat the self-assembled enzyme as only one functional protein because the copies of the enzyme are all colocalized.

### Bounded self-assembly can yield oscillations whose behavior is well-predicted by analytical formulae

#### The onset of oscillations for the bounded self-assembly system is well-predicted by two dimensionless parameters

We first consider a system where unphosphorylated kinase and phosphatase monomers self-assemble in an all-or-nothing manner into closed symmetric homomultimers. We denote by *n* the number of kinase monomers κ in the functional kinase multimer *K* and by *m* the analogous number of phosphatase monomers ρ in the multimer *P*. The reaction network is shown in Fig. 1B.

**Fig. 2:**
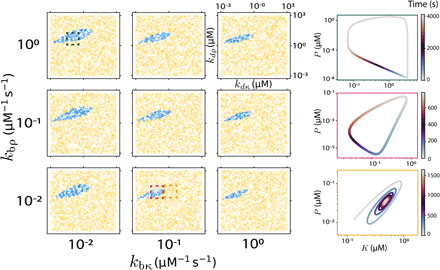
Bounded self-assembly can yield oscillations using experimentally realizable parameters. Numerical integration of eq. S1 displays parameter regimes leading to oscillations within experimental constraints. Each subplot shows the location of oscillating parameter sets as a function of *k*_*d*κ_ and *k*_*d*ρ_ for given *k*_bκ_ and *k*_bρ_; the latter two are varied for each subplot. Aside from experimental constraints (see main text for discussion), we set *n* = *m* = 2, κ_tot_ = ρ_tot_ = 10 μM, and ${\tilde{P}}_{\text{tot}}=10^{-4}$ μM. Blue points denote parameter sets leading to sustained oscillations; yellow points denote parameter sets leading to steady state. To the right of the plot, we show a few example trajectories in *K-P* phase space. The closed trajectories correspond to sustained oscillations; the final trajectory, the spiral, corresponds to a decaying oscillation and therefore to a yellow point in the figure.

Since the full equations describing this bounded self-assembly system (eq. S1) are too complex to directly tackle analytically, we numerically integrate them within the parameter ranges outlined above (section S5). Our results, shown in Fig. 2, demonstrate a significant portion of parameter space within experimental constraints capable of admitting sustained oscillations.

To simplify these equations to an analytically tractable form, we make the Michaelis-Menten approximation that enzymatic intermediates are in quasi-steady state. We then make the approximation that the enzyme and substrate concentrations are low compared to the Michaelis constants, such that the concentration of enzymatic intermediates can be entirely neglected within our analytical approximations (section S2). This approximation, like others that we will consider, is not obeyed by all oscillating solutions found numerically (Fig. 2) but is nonetheless useful in clarifying the fundamentals of a large swath of the oscillations. We find that, in contrast to well-known examples from other systems that rely on enzyme sequestration to achieve oscillations (*6*, *7*), neglecting enzyme sequestration does not preclude oscillations for our systems.

To reduce our systems further to only two differential equations, we assume a separation of timescales between the self-assembly and the enzymatic activity. In particular, we assume that phosphorylation/dephosphorylation reactions equilibrate much faster than self-assembly. The opposite separation–of–timescales limit yields oscillations only for extremely large values of *m*, which are infeasible to realize experimentally (see further discussion in section S4). We thereby arrive at the following two-dimensional system of equations

$$\begin{array}{c} \frac{\mathrm{dK}}{\mathrm{dt}}=k_{b\kappa }{\left(\frac{\kappa_{\text{tot}}-\mathrm{nK}}{1+\eta_{\kappa }\frac{K}{P+\tilde{P}}}\right)}^{n}-k_{u\kappa }K\\ \frac{\mathrm{dP}}{\mathrm{dt}}=k_{b\rho }{\left(\frac{\rho_{\text{tot}}-\mathrm{mP}}{1+\eta_{\rho }\frac{K}{P+\tilde{P}}}\right)}^{m}-k_{u\rho }P \end{array}$$ (1)

We briefly define the parameters: *k*_bκ_ is the binding rate for κ into its multimeric state, *k*_uκ_ is the respective unbinding rate, and *k*_*d*κ_ is the inverse ratio of the two; η_*K*κ_ is the specificity constant *k*_cat_/*K*_M_ for the kinase *K* acting on κ, η_*P*κ_ is the same for the phosphatase *P*, and η_κ_ = η_*K*κ_/η_*P*κ_; κ_tot_ is the total concentration of monomeric κ added to the system, a conserved quantity. Similar quantities are defined for ρ. We assume the concentration of the constitutive phosphatase $\tilde{P}>0$ throughout (see section S2).

To describe the oscillatory behavior of the system, we seek the eigenvalues of the Jacobian in the vicinity of a fixed point (*K*^⋆^, *P*^⋆^). Oscillations require coupling between the equations, motivating the approximations that in the oscillatory regime, $\eta_{\kappa }K^{⋆}\gg P^{⋆}+\tilde{P}$ (and same for η_ρ_), κ_tot_ ≫ *nK*^⋆^, and ρ_tot_ ≫ *mP*^⋆^. Defining the dimensionless concentrations $\tilde{K^{⋆}}=K^{⋆}/\tilde{P}$ and $\tilde{P^{⋆}}=P^{⋆}/\tilde{P}$, the fixed point in these limits is given by

$$\begin{array}{c} {\tilde{P^{⋆}}}^{\left(n+1\right)}=\gamma {\left(\tilde{P^{⋆}}+1\right)}^{m}\\ \tilde{K^{⋆}}=\alpha {\tilde{P^{⋆}}}^{\left(n/m\right)} \end{array}$$ (2)

where γ and α are dimensionless parameters defined by

$$\begin{array}{c} \gamma =\frac{k_{d\kappa }^{m}}{k_{d\rho }^{n+1}}{\left(\frac{\eta_{\kappa }}{\kappa_{\text{tot}}}\right)}^{\mathrm{nm}}{\left(\frac{\rho_{\text{tot}}}{\eta_{\rho }}\right)}^{(n+1)m}{\tilde{P}}^{\left(m-n-1\right)}\\ \alpha =\frac{k_{d\rho }^{n/m}}{k_{d\kappa }}{\left(\frac{\eta_{\rho }\kappa_{\text{tot}}}{\eta_{\kappa }\rho_{\text{tot}}}\right)}^{n}{\tilde{P}}^{(n-m)/m} \end{array}$$ (3)

We constrain ourselves to *m* ≤ *n* + 1 so that, within our approximations, there is no more than one physical fixed point in the system as long as $\tilde{P}>0$, simplifying our analysis. (When no solutions to Eq. 2 exist, our assumptions leading to it break down.)

Sustained oscillations in the system typically correspond to complex eigenvalues of the Jacobian with positive real parts. However, following the Poincaré-Bendixson theorem, as long as our system has a single fixed point, instability of the fixed point must imply oscillations even if they are beyond the linear regime. Translated into constraints on *P*^⋆^, instability of the fixed point corresponds to

$$(\frac{(m-1)k_{u\rho }-(n+1)k_{u\kappa }}{(n+1)k_{u\kappa }+k_{u\rho }})\frac{P^{⋆}}{\tilde{P}}>1$$ (4)

We directly verify Eq. 4 in fig. S6.

We now proceed to express Eq. 4 only in terms of the input parameters. Because Eq. 2 cannot be solved for *P*^⋆^ for general *n*, *m*, we consider the approximation that $P^{⋆}=\gamma^{1/(n+1-m)}\tilde{P}\gg \tilde{P}$, equivalent within the constraint *m* < *n* + 1 to γ ≫ 1. This approximation is most accurate for small values of *m*, since fewer terms are neglected. The approximation is motivated by the intuition that oscillations require *P* to be non-negligible compared to $\tilde{P}$; indeed, an opposite self-consistent solution, in which $P^{⋆}\ll \tilde{P}$, is incompatible with oscillations.

By simplifying Eq. 4 within this limit where $P^{⋆}=\gamma^{1/(n+1-m)}\tilde{P}$, we find that the system oscillates when

$$\nu \equiv \frac{(m-1)k_{u\rho }}{(n+1)k_{u\kappa }}>1$$ (5)

This corresponds to ensuring a positive left-hand side in Eq. 4 (see fig. S6).

**Fig. 3:**
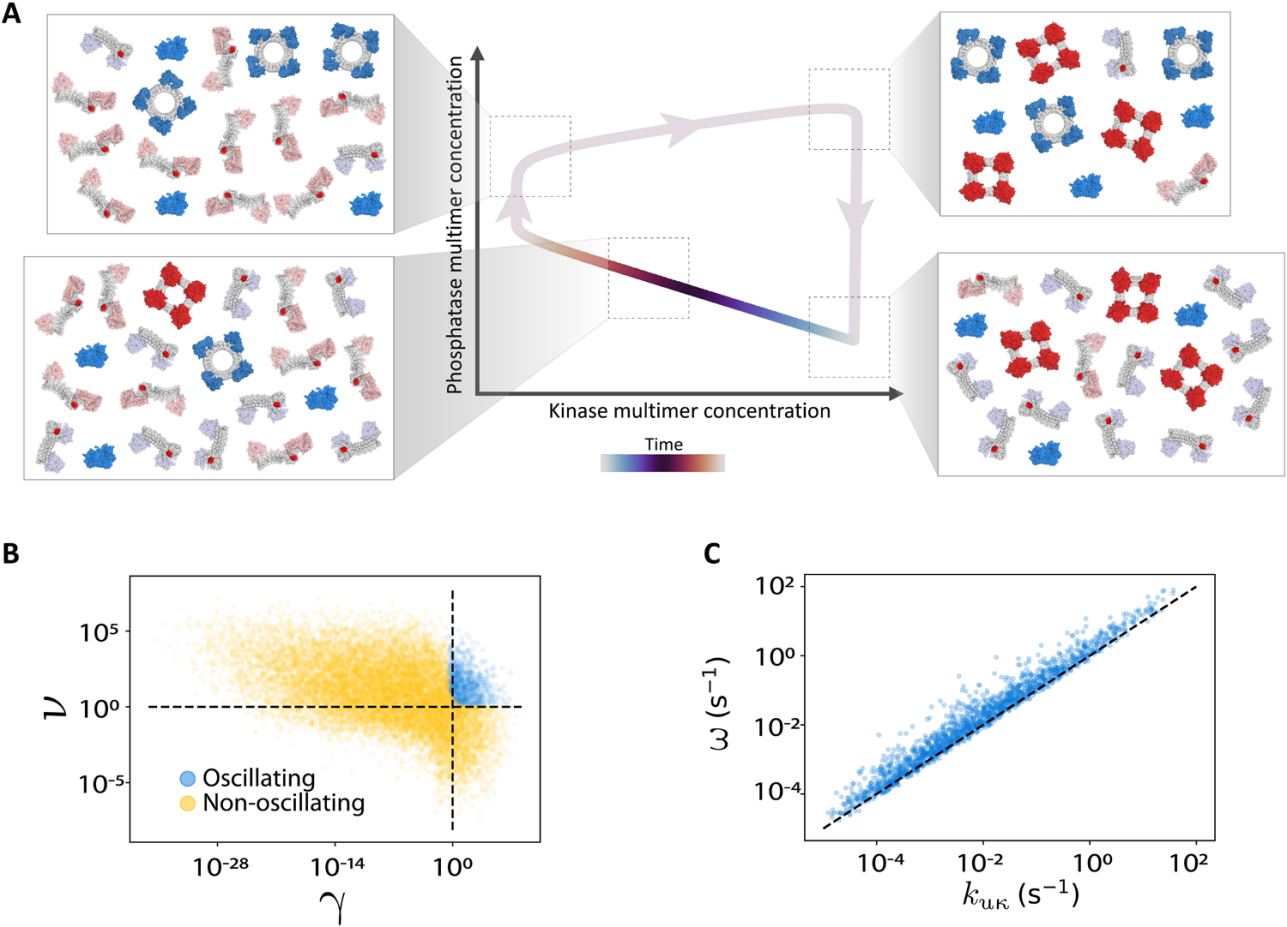
Analytical results for bounded self-assembly oscillator. In this figure, we show results from the analytically simplified bounded self-assembly oscillator (Eq. 1), using *n* = *m* = 2. (**A**) Oscillation schematic. We visualize a sample oscillation using randomly and arbitrarily chosen parameters satisfying experimental constraints. Oscillations require phosphatase multimers (blue) to dissociate faster than kinase multimers (red). The system starts with self-assembled kinases and phosphatases (top right). After rapid phosphatase disassembly and phosphorylation by the kinase multimers (bottom right), the kinases slowly disassemble, which enables the gradual dephosphorylation and self-assembly of the phosphatase monomers (bottom left). The assembled phosphatases are then able to rapidly promote their own and kinase self-assembly through dephosphorylation, returning the system to its initial state (top right). (**B**) Onset of oscillations. Numerical integration demonstrates consistency with Eq. 5 for the appearance of oscillations in the appropriate limits. Each point represents a random set of parameters, sampled within the experimentally realizable limits as described in the main text. Oscillating (blue) and non-oscillating (yellow) parameter sets can be well-separated by dimensionless combinations of parameters γ and ν. Dashed lines show where the dimensionless parameters on the axes equal unity. (**C**) Oscillation frequency. Intuition from linear stability analysis of the fixed point suggests that for the *n* = *m* = 2 system considered numerically, oscillation frequency may be determined by the dissociation rate of kinase multimers, $k_{u\kappa }$. Numerical integration demonstrates that $k_{u\kappa }$ is indeed highly predictive of oscillation frequency (*R*^2^ ≈ 0.66; *R*^2^ ≈ 0.93 in log space) and underestimates the true frequency by a typical factor of ~4. Black dashed line shows ω = *k*_uκ_.

 The implication of Eq. 5, that oscillations require a larger dissociation rate of the phosphatase monomers compared to the kinase monomers (at least, for *n* + 2 ≥ *m*), agrees with intuition found by visualizing individual oscillation cycles (Fig. 3A). In *K-P* space, oscillations proceed in a counterclockwise fashion: Starting from the unphosphorylated state, the monomers self-assemble into multimers (top right). The larger dissociation rate of phosphatase multimers leads those to dissociate first and get phosphorylated by the abundant kinase multimers (bottom right). Next, the kinase multimers slowly dissociate, enabling the gradual dephosphorylation and self-assembly of the phosphatase monomers by the constitutive and self-assembled phosphatases (bottom left). Once kinase multimer levels have decreased and enough phosphatase multimers have formed, the latter quickly dephosphorylate the remaining monomers (top left), and the system returns to its initial state (top right).

We verify that the approximate formula given by Eq. 5 is valid in describing Eq. 1 by comparing it to oscillations found by random parameter searches in Fig. 3B. We numerically integrate Eq. 1 with random parameters chosen to satisfy the experimental constraints described previously (including setting η_κ_ = η_ρ_) and with *n* = *m* = 2. We constrain concentrations κ_tot_ and ρ_tot_ to be within 10^−3^ to 10^2^ μM, while we set the bounds of $\tilde{P}$ to 10^−8^ and 10^−2^ μM. For each parameter set, we numerically estimate the fixed point using Python’s scipy.optimize.root function. We only show parameter sets estimated to agree with the approximations described before Eq. 2 (with >5× substituted for ≫). We found no oscillations in ∼2.5 × 10^4^ parameter sets for which η_K_ or η_P_ is less than $(P^{⋆}+\tilde{P})/K^{⋆}$. Each blue (yellow) point in the figure corresponds to a single parameter set found to produce (not produce) oscillations starting from initial conditions of (*K*, *P*) = (0,0). Oscillations are almost exclusively found in the quadrant γ > 1, ν > 1. Values of γ slightly less than unity are also found to produce oscillations, as shown in the figure.

These results show that oscillations are found when the dissociation rate of the phosphatase multimer is in a middle range between two extremes. To see oscillations, the phosphatase multimer must dissociate significantly faster than the kinase multimer (ν > 1); however, the dissociation rate must concurrently be small enough such that the fixed point concentration of phosphatase multimer is larger than the constitutive phosphatase concentration (γ > 1). In contrast to intuition from other systems, which signifies that higher-order nonlinearities increase the parameter range producing oscillations (*6*), here, we found that more nonlinear self-assembly (i.e., higher values of *n* and *m*) makes oscillations less frequent. We find that oscillations are robust to even order-of-magnitude variations in most other parameters (section S3 and fig. S2).

Our results suggest that oscillations in the concentrations of unphosphorylated monomers or of phosphatase multimers may be most straightforward to visualize experimentally, as these concentrations typically vary by several orders of magnitude across an oscillatory cycle (fig. S8). Parameter sets yielding largest oscillation amplitudes are also typically farthest from the bifurcation point (fig. S10).

#### The frequency of resulting oscillations can be well-predicted by assuming that kinase multimer dissociation is rate limiting

We next seek to predict how system parameters tune the frequency of resulting oscillations when they appear. Within the linear regime around the fixed point, in the limit of Eq. 5, the frequency of oscillations ω is predicted to be

$$\omega_{\text{pred}}^{2}=-\frac{1}{4}{\left[(n+1)k_{u\kappa }+(m-1)k_{u\rho }\right]}^{2}+\mathrm{nm}k_{u\kappa }k_{u\rho }$$ (6)

While Eq. 6 agrees well with the true squared frequency for those parameter sets where it is positive, oscillations are frequently found in the nonlinear regime in which it is not applicable (fig. S7A). However, the intuition given by Eq. 6, that oscillation frequency is determined by the unbinding rates of the kinase and phosphatase multimers, may still be valid outside the linear regime of the fixed point. This intuition is reasonable given the separation–of–timescales limit in which we are operating, of enzymatic reactions equilibrating much faster than self-assembly. Since oscillations for the *n* = *m* = 2 system that we considered numerically require that *k*_uρ_ > *k*_uκ_ (i.e., ν > 1), Eq. 6 implies that the limiting reaction in the oscillations is the unbinding of the kinase multimers. To test that implication, we compare *k*_uκ_ to the frequency of oscillations found through numerical integration in Fig. 3C. (We make no constraints on the fixed points of the parameter sets considered here.) We find a strong correlation (*R*^2^ = 0.66; *R*^2^ = 0.93 in log space) and a root mean square relative error of ∼3.9, demonstrating that *k*_uκ_ can accurately predict the frequency of oscillations in this system.

Oscillations found within experimental constraints for Eq. 1 have periods ranging from fractions of a second to >1 day (fig. S7B). For oscillations found for the full system of equations plotted in Fig. 2, we find periods within a slightly more constrained range than for the simplified system but still spanning orders of magnitude, between ~1 min and >1 day.

### Unbounded self-assembly can yield oscillations within experimental constraints in the limit of fast self-assembly compared to enzymatic activity

**Fig. 4:**
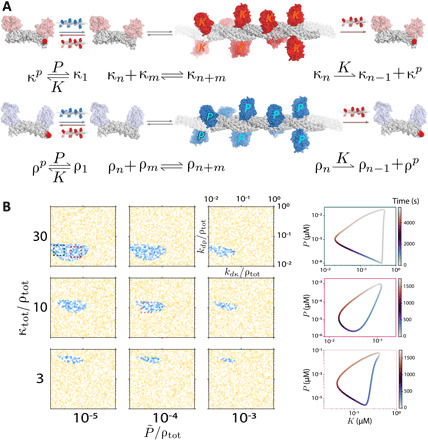
Unbounded self-assembly oscillator. (**A**) Unbounded self-assembly reactions. We consider a related system to that shown in Fig. 1 but relying on kinase and phosphatase monomers that self-assemble into unbounded fibers of arbitrary length. In addition, we assume that the final monomer of each fiber can get phosphorylated by a kinase multimer, at which point it can no longer rejoin the fiber until it is dephosphorylated. (**B**) Unbounded self-assembly oscillations using experimentally realizable parameters. Numerical integration of Eq. 9 displays parameter regimes leading to oscillations within experimental constraints. Eq. 9 was used in place of the full system of equations (eq. S8) because of the infinite dimensionality of the latter. ρ_tot_ sets the concentration scale.

We now consider a second system in which individual species κ and ρ can self-assemble incrementally into one-dimensional unbounded fibers (Fig. 4A). Unlike the bounded case in which no phosphorylation sites are accessible in the multimeric state, in this system, one is (corresponding to the final protein in the fiber). An *n*-mer of species *X* (where *X* is either κ or ρ), *X_n_*, can be created either from binding two smaller molecules *X_m_* and *X*_*n*−*m*_ or from the spontaneous breaking of a bond of a larger molecule. The concentration of *X_n_* decreases when an *X_n_* molecule either binds to any other molecule or breaks any of its *n −* 1 bonds. The equations for self-assembly of species *X* are therefore given by

$$\begin{array}{c} \frac{{\mathrm{dX}}_{n}}{\mathrm{dt}}=k_{bX}(\overset{n-1}{\underset{m=1}{\Sigma }}X_{m}X_{n-m}-2X_{n}\overset{\infty }{\underset{m=1}{\Sigma }}X_{m})\\ +k_{uX}(2\overset{\infty }{\underset{m=n+1}{\Sigma }}X_{m}-(n-1)X_{n}) \end{array}$$ (7)

As in the first system, each protein of type κ (ρ) includes a split kinase (phosphatase). While in the bounded system, enzymes required exactly *n* or *m* monomers to self-assemble, here, an enzyme is created by any group of more than one monomer (i.e., *X_n_* is a functional enzyme as long as *n* ≥ 2). We assume that when a multimer is phosphorylated, its final monomer dissociates from the fiber and cannot reassociate in its phosphorylated state. A less stringent assumption, that the phosphorylated monomer does not dissociate automatically but merely prevents new monomers from binding to that end of the molecule, appears to be incompatible with oscillations, at least in both separation–of–timescales limits.

The full equations describing the system are given in the Supplementary Materials (eq. S8). As previously, we search for a two-dimensional set of simplified equations by considering a separation of timescales between enzymatic reactions and self-assembly, along with the same simplifying assumptions as considered for the bounded self-assembly system (eq. S9). The limit considered for the first system, of fast phosphorylation/dephosphorylation compared to self-assembly, would disallow multimers from forming in this system, since, here, phosphorylation is accompanied by dissociation of the final multimer in the chain. Therefore, the separation–of–timescales limit considered in the bounded self-assembly system is no longer applicable for this system. Instead, we consider the opposite limit, of fast self-assembly compared to enzymatic activity. At steady state, *X_n_* is given by

$$X_{n}=\frac{k_{uX}}{k_{bX}}{\left(\frac{x}{1+x+\sqrt{1+2x}}\right)}^{n}$$ (8)

where *x* = 2*k*_b*X*_*X*_tot_/*k*_u*X*_ = 2*X*_tot_/*k_dX_*. The same steady state is reached even if self-assembly involves binding and unbinding only a single monomer at a time.

The concentrations of phosphorylated monomers as a function of time are given by κ*^p^* and ρ*^p^*. The total amount of kinase present is given by $K=\Sigma_{i=2}^{\infty }\kappa_{i}$ and similarly for phosphatase. Since only the phosphorylation site of the final monomer in a multimer is exposed, the total number of available phosphorylation sites in the κ species is given by $\Sigma_{i=1}^{\infty }\kappa_{i}$ (and similarly for ρ). The system can be described by two differential equations for *k* = 2(κ_tot_ − κ*^p^*)/*k*_*d*κ_ and *p* = 2(ρ_tot_ − ρ*^p^*)/*k*_*d*ρ_

$$\begin{array}{c} \frac{\mathrm{dk}}{\mathrm{dt}}=\frac{-2\eta_{K\kappa }k}{1+\sqrt{1+2k}}K+\eta_{P\kappa }(\frac{2\kappa_{\text{tot}}}{k_{d\kappa }}-k)(P+\tilde{P})\\ \frac{\mathrm{dp}}{\mathrm{dt}}=\frac{-2\eta_{K\rho }p}{1+\sqrt{1+2p}}K+\eta_{P\rho }(\frac{2\rho_{\text{tot}}}{k_{d\rho }}-p)(P+\tilde{P})\\ K=k_{d\kappa }\frac{k^{2}}{\left(1+\sqrt{1+2k})(1+k+\sqrt{1+2k}\right)}\\ P=k_{d\rho }\frac{p^{2}}{\left(1+\sqrt{1+2p})(1+p+\sqrt{1+2p}\right)} \end{array}$$ (9)

Unlike in the previous system for which the frequency and onset of oscillations can be determined by simple formulae by taking a limit of the two-dimensional system, no such limits give similarly straightforward results for Eq. 9. Instead, we analyze Eq. 9 through random parameter searches (Fig. 4B). As shown in the figure, we find that although these equations assume an opposite separation–of–timescales limit to that yielding experimentally realizable oscillations for the system of bounded self-assembly, they can nevertheless yield oscillations within a significant region of parameter space consistent with experimental constraints.

We find that increased values of κ_tot_/ρ_tot_ and decreased values of $\tilde{P}/\rho_{\text{tot}}$ lead to more robust oscillations in this system; see fig. S3 for further discussion of oscillation robustness. The periods of oscillations found ranged from <1 min to >1 day (fig. S7B). Finally, we find that the concentrations of phosphatase multimers and unphosphorylated phosphatase monomers typically vary by several orders of magnitude across an oscillatory cycle (fig. S9), suggesting that oscillations may be most straightforward to visualize experimentally by measuring these concentrations.

## DISCUSSION

In summary, we have presented two posttranslational protein–based oscillators motivated by the biological KaiABC system and by the synthetic dual-feedback genetic oscillator. Both systems that we present rely on split kinase and phosphatase self-assembling to form functional enzymes and on that self-assembly being inhibited by phosphorylation of the split monomers. The two systems differ mainly in the nature of the self-assembly as all-or-nothing into bounded structures of specified size or incremental into unbounded one-dimensional fibers.

Both systems are capable of producing oscillations within experimental constraints, using experimentally determined wild-type values for kinase and phosphatase activity and for a range of designed self-assembly rates. We have shown that neither complex reactions nor large number of species are necessary to achieve oscillations: Both networks that we present use only three protein species interacting only through reversible binding and phosphorylation/dephosphorylation reactions, and the resulting oscillations can be understood as arising from a minimal system of two differential equations in both cases.

Although the systems that we described shared much in common, they produced robust oscillations in opposite separation–of–timescales limits from one another: The first primarily oscillates when self-assembly is much slower than enzymatic reactions; the second when it is much faster. These two networks are thus complementary: Depending on the parameter regime most easily accessible to an experimentalist, one or the other network might be preferable to implement. Nevertheless, the conditions giving rise to oscillations shared similarities in the two systems. Smaller values of constitutive phosphatase (equivalent to larger values of γ in the case of bounded self-assembly) lead to more robust oscillations in both systems, as do smaller values of *k*_*d*κ_ (i.e., larger values of ν).

Our work paves the way toward the rational design and experimental realization of protein-based far-from-equilibrium dynamic systems. The models described here were designed to be feasible to synthesize experimentally and are guiding an implementation in the test tube that is currently under way.

## Supplementary Material

http://advances.sciencemag.org/cgi/content/full/6/51/eabc1939/DC1

Adobe PDF - abc1939_SM.pdf

Self-asembly–based posttranslational protein oscillators

## SUPPLEMENTARY MATERIALS

Supplementary material for this article is available at http://advances.sciencemag.org/cgi/content/full/6/51/eabc1939/DC1

## Appendix

View/request a protocol for this paper from *Bio-protocol*.
