# Supplementary material for: Self-assembly–based posttranslational protein oscillators
Source: Sci Adv. 2020 Dec 16;6(51):eabc1939. doi: 10.1126/sciadv.abc1939 (PMC7744077; doi:10.1126/sciadv.abc1939)
Supplement: http://advances.sciencemag.org/cgi/content/full/6/51/eabc1939/DC1 [file supp_6_51_eabc1939__index.html]

Science Advances | Science AdvancesAAASSearchScience AdvancesMenu

## Supplementary Materials

# Self-assemblyï¿½based posttranslational protein oscillators

Ofer Kimchi, Carl P. Goodrich, Alexis Courbet, Agnese I. Curatolo, Nicholas B. Woodall, David Baker, Michael P. Brenner

Download Supplement

**This PDF file includes:**

- Sections S1 to S6
- Figs. S1 to S10
- Table S1
- References

**Files in this Data Supplement:**

- Adobe PDF - abc1939\_SM.pdf
