# Supplementary material for: Self-assembly–based posttranslational protein oscillators
Source: Sci Adv. 2020 Dec 16;6(51):eabc1939. doi: 10.1126/sciadv.abc1939 (PMC7744077; doi:10.1126/sciadv.abc1939)
Supplement: http://advances.sciencemag.org/cgi/content/full/6/51/eabc1939/DC1 [file supp_6_51_eabc1939__1.pdf]

Science Advances
AAAS

advances.sciencemag.org/cgi/content/full/6/51/eabc1939/DC1

Supplementary Materials for

Self-assembly–based posttranslational protein oscillators

Ofer Kimchi*, Carl P. Goodrich, Alexis Courbet, Agnese I. Curatolo, Nicholas B. Woodall, David Baker, Michael P. Brenner

*Corresponding author. Email: okimchi@g.harvard.edu

Published 16 December 2020, *Sci. Adv.* **6**, eabc1939 (2020)
DOI: 10.1126/sciadv.abc1939

This PDF file includes:

Sections S1 to S6
Figs. S1 to S10
Table S1
References

Supplementary Material

S1 Other oscillation schemes attempted

Before trying self-assembly based oscillations, we tried implementing oscillations based on phosphorylations or binding events accompanying a conformational change in the molecule. Such conformational changes can be difficult to design, but the recently-published LOCKR system (*28, 29*) demonstrates one way in which binding can accompany a conformational change. We considered a molecule A which can be phosphorylated or bind to another molecule. We assume that when it is bound or phosphorylated, the molecule undergoes a conformational change; in the language of the LOCKR system, it opens. We assume that the rate of binding of any molecule to the closed state $A^\star$ can be smaller than the analogous binding rate to the open state, but no other asymmetries between the rates of analogous reactions are allowed. We did not make simplifying assumptions such as the Michaelis-Menten approximation when considering these systems.

We found oscillations are possible if A can bind, and thus sequester, free kinases (Fig. S1a). Oscillations are also possible if A can bind a separate "key" peptide b, which itself either binds free kinase (K) or phosphatase (P) molecules. Finally, oscillations can also be found if b, either alongside or instead of binding kinase or phosphatase, can itself get phosphorylated. We assume phosphorylated b is inert, except in that it can interact with phosphatase to get dephosphorylated (Fig. S1b). However, we found no evidence of possible oscillations within the experimental limits considered in this paper, after trying for each network 2×10^6 random parameter sets logarithmically distributed within the acceptable ranges.

For example, although experimentally realizable values of η_κ and η_ρ are near (slightly above) unity, we found no evidence of oscillations for the system shown in Fig. S1a with values of $\eta_\kappa =$

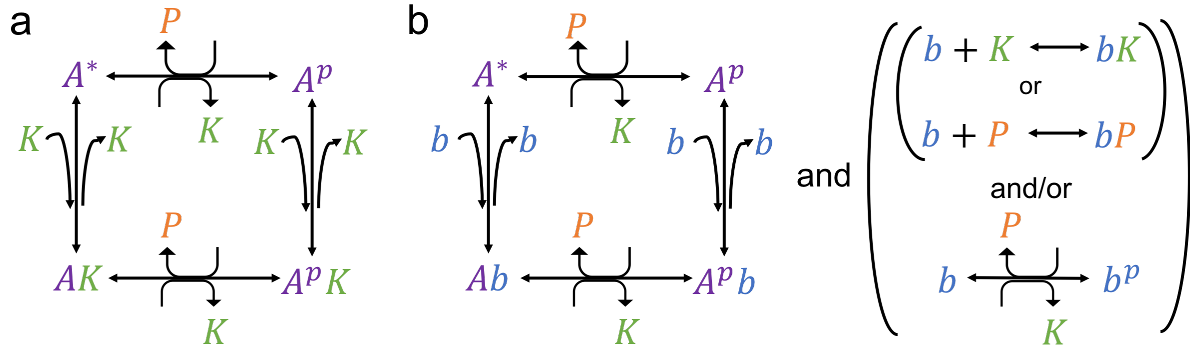

Figure S1: **Reaction networks giving oscillations outside of experimentally realizable regime.** See text for discussion.

η_ρ greater than 0.02, a difference of two orders of magnitude from the experimentally realizable regime. With future advances in protein engineering, the realm of experimental realizability may well expand and these networks may be able to yield *in vitro* oscillations. However, our work here is focused on those parameters that are currently reasonably accessible in the lab as described in the main text, and we therefore do not consider the networks shown in Fig. S1 further.

S2 Full kinetic equations and derivations of main text equations

Bounded self-assembly

We denote the concentration of phosphorylated (monomeric) κ by κ^p (and similarly for ρ). The concentration of the enzyme-substrate complex comprised of κ and K bound is denoted $\kappa \cdot K$. Binding, unbinding, and catalytic rate constants for the enzyme-substrate complexes are given by $k_{bK\kappa}, k_{uK\kappa}$, and $k_{cK\kappa}$, respectively. We use similar conventions for all other enzyme-substrate complexes. The full equations for the first system are:

$$
\begin{aligned}
\frac{d\kappa}{dt} &= -n(k_{b\kappa}\kappa^n + k_{u\kappa}K) - k_{bK\kappa}\kappa K + k_{uK\kappa}\kappa\cdot K + k_{cP\kappa}(\kappa^p\cdot P + \kappa^p\cdot\tilde{P}) \\
\frac{d\rho}{dt} &= -m(k_{b\rho}\rho^m + k_{u\rho}P) - k_{bK\rho}\rho K + k_{uK\rho}\rho\cdot K + k_{cP\rho}(\rho^p\cdot P + \rho^p\cdot\tilde{P}) \\
\frac{dK}{dt} &= k_{b\kappa}\kappa^n - k_{u\kappa}K - k_{bK\kappa}\kappa K + (k_{uK\kappa} + k_{cK\kappa})\kappa\cdot K - k_{bK\rho}\rho K + (k_{uK\rho} + k_{cK\rho})\rho\cdot K \\
\frac{dP}{dt} &= k_{b\rho}\rho^m - k_{u\rho}P - k_{bP\kappa}\kappa^p P + (k_{uP\kappa} + k_{cP\kappa})\kappa^p\cdot P - k_{bP\rho}\rho^p P + (k_{uP\rho} + k_{cP\rho})\rho\cdot P \\
\frac{d\kappa^p}{dt} &= -k_{bP\kappa}\kappa^p(P+\tilde{P}) + k_{uP\kappa}(\kappa^p\cdot P + \kappa^p\cdot\tilde{P}) + k_{cK\kappa}\kappa\cdot K \\
\frac{d\rho^p}{dt} &= -k_{bP\rho}\rho^p(P+\tilde{P}) + k_{uP\rho}(\rho^p\cdot P + \rho^p\cdot\tilde{P}) + k_{cK\rho}\rho\cdot K \\
\frac{d\kappa\cdot K}{dt} &= k_{bK\kappa}\kappa K - (k_{uK\kappa} + k_{cK\kappa})\kappa\cdot K \\
\frac{d\rho\cdot K}{dt} &= k_{bK\rho}\rho K - (k_{uK\rho} + k_{cK\rho})\rho\cdot K \\
\frac{d\kappa^p\cdot P}{dt} &= k_{bP\kappa}\kappa^p P - (k_{uP\kappa} + k_{cP\kappa})\kappa^p\cdot P \\
\frac{d\rho^p\cdot P}{dt} &= k_{bP\rho}\rho^p P - (k_{uP\rho} + k_{cP\rho})\rho^p\cdot P \\
\frac{d\kappa^p\cdot\tilde{P}}{dt} &= k_{bP\kappa}\kappa^p\tilde{P} - (k_{uP\kappa} + k_{cP\kappa})\kappa^p\cdot\tilde{P} \\
\frac{d\rho^p\cdot\tilde{P}}{dt} &= k_{bP\rho}\rho^p\tilde{P} - (k_{uP\rho} + k_{cP\rho})\rho^p\cdot\tilde{P} \\
\frac{d\tilde{P}}{dt} &= -k_{bP\kappa}\kappa^p\tilde{P} + (k_{uP\kappa} + k_{cP\kappa})\kappa^p\cdot\tilde{P} - k_{bP\rho}\rho^p\tilde{P} + (k_{uP\rho} + k_{cP\rho})\rho\cdot\tilde{P}.
\end{aligned}
\tag{S1}
$$

Making only the Michaelis-Menten approximation for enzymatic reactions and accounting for conservation laws, the equations can be reduced to the following four-dimensional system of equations:

$$
\begin{aligned}
\frac{dK}{dt} &= k_{b\kappa}\kappa^n - k_{u\kappa}K \\
\frac{dP}{dt} &= k_{b\rho}\rho^m - k_{u\rho}P \\
\frac{d\kappa}{dt} &= -n(k_{b\kappa}\kappa^n - k_{u\kappa}K) - \eta_{K\kappa}\kappa K + \eta_{P\kappa}\kappa^p(P+\tilde{P}) \\
\frac{d\rho}{dt} &= -m(k_{b\rho}\rho^m - k_{u\rho}P) - \eta_{K\rho}\rho K + \eta_{P\rho}\rho^p(P+\tilde{P}) \\
\kappa_{\text{tot}} &= \kappa + \kappa^p + nK + (n+1)\frac{\kappa K}{K_{M_{K\kappa}}} + \frac{\kappa^p(P+\tilde{P})}{K_{M_{P\kappa}}} + n\frac{\rho K}{K_{M_{K\rho}}} \\
\rho_{\text{tot}} &= \rho + \rho^p + mP + \frac{\rho K}{K_{M_{K\rho}}} + (m+1)\frac{\rho^p P}{K_{M_{P\rho}}} + \frac{\rho^p \tilde{P}}{K_{M_{P\rho}}} + m\frac{\kappa^p P}{K_{M_{P\kappa}}}
\end{aligned}
\tag{S2}
$$

where as in the main text, $\eta_{K\kappa}$ is the specificity constant $k_{cK\kappa}k_{bK\kappa}/(k_{uK\kappa} + k_{cK\kappa}) = k_{cK\kappa}/K_{M_{K\kappa}}$, and similar constants are similarly defined for other reactions.

In order to reduce our system further to only two differential equations, we assume a separation of timescales between the self-assembly and the enzymatic activity. In particular, we assume that phosphorylation/dephosphorylation reactions equilibrate much faster than self-assembly; see Section S4 for consideration of the opposite limit. Within the Michaelis-Menten approximation, this can be written as:

$$
\begin{aligned}
\eta_{K\kappa}\kappa K &= \eta_{P\kappa}\kappa^p(P+\tilde{P}) \\
\eta_{K\rho}\rho K &= \eta_{P\rho}\rho^p(P+\tilde{P})
\end{aligned}
\tag{S3}
$$

Making the approximation that the Michaelis constants are large compared to concentrations of the various components (such that $\kappa_{\text{tot}} = \kappa + \kappa^p + nK$, and likewise for ρ_{tot}) we arrive at

$$
\begin{aligned}
\kappa &= \frac{\kappa_{\text{tot}} - nK}{1 + \eta_\kappa \frac{K}{P+\tilde{P}}} \\
\rho &= \frac{\rho_{\text{tot}} - mP}{1 + \eta_\rho \frac{K}{P+\tilde{P}}}
\end{aligned}
\tag{S4}
$$

which, in conjunction with Eqn. S2, leads to Eqn. 1.

Bounded self-assembly: Evaluating the trace of the Jacobian

In order to derive Eqn. 4, we start by computing the Jacobian of Eqn. 1 and taking its trace. The trace can then be simplified by making the same approximations made to arrive at Eqn. 2 (namely, that $\eta_\kappa K^\star \gg P^\star + \tilde{P}$, $\eta_\rho K^\star \gg P^\star + \tilde{P}$, $\kappa_{\text{tot}} \gg nK^\star$, and $\rho_{\text{tot}} \gg mP^\star$). It is useful at this point to use Eqn. 2 to express the trace entirely in terms of $P^\star$.

$$\begin{aligned}\text{tr}(J) =& \frac{k_{d\kappa}}{\eta_\rho} k_{u\rho} m P^\star \rho_{\text{tot}} \left(\frac{\eta_\kappa \rho_{\text{tot}}}{\eta_\rho \kappa_{\text{tot}}}\right)^n (k_{d\rho} P^\star)^{-\frac{n+1}{m}} - \\ & \left(\frac{k_{b\kappa} n^2 k_{d\rho}^{\frac{n}{m}} P^{\star(n/m)} \left(\frac{\eta_\rho \kappa_{\text{tot}}}{\eta_\kappa \rho_{\text{tot}}}\right)^n}{\kappa_{\text{tot}}} + (n+1)k_{u\kappa} + \frac{k_{u\rho} m^2 P^\star}{\rho_{\text{tot}}} + k_{u\rho}\right).\end{aligned} \tag{S5}$$

We now replace the factors of $P^{\star\left(-\frac{n+1}{m}\right)}$ and $P^{\star(n/m)}$ with their appropriate expressions given Eqn. 2, getting a far cleaner expression:

$$\text{tr}(J) = -(n+1)k_{u\kappa} - n^2 k_{u\kappa} \frac{K^\star}{\kappa_{\text{tot}}} - k_{u\rho} + m k_{u\rho} \left(\frac{P^\star}{P^\star + \tilde{P}} - m\frac{P^\star}{\rho_{\text{tot}}}\right). \tag{S6}$$

After factoring out $nk_{u\kappa}$, we can rely on the approximations made previously that $\kappa_{\text{tot}} \gg nK^\star$, and $\rho_{\text{tot}} \gg mP^\star$ to neglect the second and final terms, leading to

$$\text{tr}(J) = -(n+1)k_{u\kappa} - k_{u\rho} + m k_{u\rho} \frac{P^\star}{P^\star + \tilde{P}} \tag{S7}$$

which we set greater than zero to arrive at Eqn. 4.

The $\tilde{P} = 0$ case

Eqns. S2 demonstrate that if $\tilde{P} = 0$, a new fixed point appears at $\kappa = \rho = K = P = 0$. The Jacobian at that fixed point has two negative and two zero eigenvalues. We find no evidence of oscillations in Eqns. S1 with $\tilde{P} = 0$ in 5×10^4 random parameter sets, nor do we find any oscillations when plotting the analogue of Fig. 2 for the case of $\tilde{P} = 0$.

Intuitively, if a system has no constitutive phosphatase activity, then if any fluctuation brings the total number of dephosphorylated ρ monomers to a value less than m, no recovery of phosphorylation activity is possible. In the presence of constitutive phosphatase, this is no longer true, leading to oscillation robustness. We therefore assume $\tilde{P} > 0$ throughout the manuscript.

Unbounded self-assembly

The full equations describing the second system are:

$$
\begin{aligned}
\frac{d\kappa_n}{dt} &= k_{b\kappa}\left(\sum_{m=1}^{n-1}\kappa_m\kappa_{n-m} - 2\kappa_n\sum_{m=1}^{\infty}\kappa_m\right) + k_{u\kappa}\left(2\sum_{m=n+1}^{\infty}\kappa_m - (n-1)\kappa_n\right)\\
&\quad + \sum_{m=2}^{\infty}\left(k_{cK\kappa}\kappa_{n+1}\cdot\kappa_m - k_{bK\kappa}\kappa_n\kappa_m + k_{uK\kappa}\kappa_n\cdot\kappa_m\right) + \delta_{n,1}k_{cP\kappa}\left(\kappa^p\cdot\tilde{P} + \sum_{m=2}^{\infty}\kappa^p\cdot\rho_m\right)\\
\frac{d\rho_n}{dt} &= k_{b\rho}\left(\sum_{m=1}^{n-1}\rho_m\rho_{n-m} - 2\rho_n\sum_{m=1}^{\infty}\rho_m\right) + k_{u\rho}\left(2\sum_{m=n+1}^{\infty}\rho_m - (n-1)\rho_n\right)\\
&\quad + \sum_{m=2}^{\infty}\left(k_{cK\rho}\rho_{n+1}\cdot\kappa_m - k_{bK\rho}\rho_n\kappa_m + k_{uK\rho}\rho_n\cdot\kappa_m\right) + \delta_{n,1}k_{cP\rho}\left(\rho^p\cdot\tilde{P} + \sum_{m=2}^{\infty}\rho^p\cdot\rho_m\right)\\
\frac{d\tilde{P}}{dt} &= -k_{bP\kappa}\kappa^p\tilde{P} + (k_{uP\kappa}+k_{cP\kappa})\kappa^p\cdot\tilde{P} - k_{bP\rho}\rho^p\tilde{P} + (k_{uP\rho}+k_{cP\rho})\rho^p\cdot\tilde{P}\\
\frac{d\kappa^p}{dt} &= k_{cK\kappa}\sum_{n=1}^{\infty}\sum_{m=2}^{\infty}\kappa_n\cdot\kappa_m - k_{bP\kappa}\kappa^p\left(\tilde{P} + \sum_{m=2}^{\infty}\rho_m\right) + k_{uP\kappa}\left(\kappa^p\cdot\tilde{P} + \sum_{m=2}^{\infty}\kappa^p\cdot\rho_m\right)\\
\frac{d\rho^p}{dt} &= k_{cK\rho}\sum_{n=1}^{\infty}\sum_{m=2}^{\infty}\rho_n\cdot\kappa_m - k_{bP\rho}\rho^p\left(\tilde{P} + \sum_{m=2}^{\infty}\rho_m\right) + k_{uP\rho}\left(\rho^p\cdot\tilde{P} + \sum_{m=2}^{\infty}\rho^p\cdot\rho_m\right)\\
\frac{d\kappa_n\cdot\kappa_m}{dt} &= k_{bK\kappa}\kappa_n\kappa_m - (k_{uK\kappa}+k_{cK\kappa})\kappa_n\cdot\kappa_m\\
\frac{d\rho_n\cdot\kappa_m}{dt} &= k_{bK\kappa}\kappa_n\kappa_m - (k_{uK\kappa}+k_{cK\kappa})\kappa_n\cdot\kappa_m\\
\frac{d\kappa^p\cdot\rho_m}{dt} &= k_{bP\kappa}\kappa^p\rho_m - (k_{uP\kappa}+k_{cP\kappa})\kappa^p\cdot\rho_m\\
\frac{d\kappa^p\cdot\tilde{P}}{dt} &= k_{bP\kappa}\kappa^p\tilde{P} - (k_{uP\kappa}+k_{cP\kappa})\kappa^p\cdot\tilde{P}\\
\frac{d\rho^p\cdot\rho_m}{dt} &= k_{bP\rho}\rho^p\rho_m - (k_{uP\rho}+k_{cP\rho})\rho^p\cdot\rho_m\\
\frac{d\rho^p\cdot\tilde{P}}{dt} &= k_{bP\rho}\rho^p\tilde{P} - (k_{uP\rho}+k_{cP\rho})\rho^p\cdot\tilde{P}.
\end{aligned}
\tag{S8}
$$

Within the Michaelis-Menten approximation and after accounting for conservation laws, these equations become:

$$
\begin{aligned}
\frac{d\kappa_n}{dt} &= k_{b\kappa}\left(\sum_{m=1}^{n-1}\kappa_m\kappa_{n-m} - 2\kappa_n\sum_{m=1}^{\infty}\kappa_m\right) + k_{u\kappa}\left(2\sum_{m=n+1}^{\infty}\kappa_m - (n-1)\kappa_n\right) \\
&\quad + \eta_{K\kappa}(\kappa_{n+1}-\kappa_n)K + \delta_{n,1}\eta_{P\kappa}\kappa^p(P+\tilde{P}) \\
\frac{d\rho_n}{dt} &= k_{b\rho}\left(\sum_{m=1}^{n-1}\rho_m\rho_{n-m} - 2\rho_n\sum_{m=1}^{\infty}\rho_m\right) + k_{u\rho}\left(2\sum_{m=n+1}^{\infty}\rho_m - (n-1)\rho_n\right) \\
&\quad + \eta_{K\rho}(\rho_{n+1}-\rho_n)K + \delta_{n,1}\eta_{P\rho}\rho^p(P+\tilde{P}) \\
K &= \sum_{n=2}^{\infty}\kappa_n; \quad P = \sum_{n=2}^{\infty}\rho_n \\
\kappa_{\text{tot}} &= \kappa^p + \sum_{n=1}^{\infty}n\kappa_n + \sum_{n=2}^{\infty}\kappa_n\sum_{m=1}^{\infty}\left(\frac{(n+m)\kappa_m}{K_{M_{K\kappa}}} + \frac{n\rho_m}{K_{M_{K\rho}}}\right) + \frac{\kappa^p(P+\tilde{P})}{K_{M_{P\kappa}}} \\
\rho_{\text{tot}} &= \rho^p + \sum_{n=1}^{\infty}n\rho_n + \sum_{n=2}^{\infty}\kappa_n\sum_{m=1}^{\infty}\frac{m\rho_m}{K_{M_{K\rho}}} + \sum_{n=2}^{\infty}\rho_n\left(\frac{n\kappa^p}{K_{M_{P\kappa}}} + \frac{(n+1)\rho^p}{K_{M_{P\rho}}}\right) + \frac{\rho^p\tilde{P}}{K_{M_{P\rho}}}.
\end{aligned}
\tag{S9}
$$

In order to arrive at Eqn. 9, we assume a separation of timescales between the self-assembly and the enzymatic activity. In particular, we assume that self-assembly reactions equilibrate much faster than phosphorylation/dephosphorylation. This is the opposite separation-of-timescales limit to that considered in the bounded self-assembly case. The reason we consider this limit here is that the limit of fast enzymatic activity compared to self-assembly (the limit considered for the bounded system) would result in any multimers immediately being broken up into monomers, since phosphorylation in this system is accompanied by the final monomer in the chain dissociating from the multimer.

We can then write the dynamics of the system only in terms of the phosphorylated monomers κ^p and ρ^p.

$$
\begin{aligned}
\frac{d\kappa^p}{dt} &= \eta_{K\kappa}\sum_{n=1}^{\infty}\kappa_n K - \eta_{P\kappa}\kappa^p(P+\tilde{P}) \\
\frac{d\rho^p}{dt} &= \eta_{K\rho}\sum_{n=1}^{\infty}\rho_n K - \eta_{P\rho}\rho^p(P+\tilde{P})
\end{aligned}
\tag{S10}
$$

where K and P are as defined in Eqn. 9. Writing these equations in terms of $k = 2(\kappa_{\text{tot}} - \kappa^p)/k_{d\kappa}$ and $p = 2(\rho_{\text{tot}} - \rho^p)/k_{d\rho}$, we arrive at Eqn. 9.

S3 Robustness analysis

Here we describe the robustness of oscillations to errors in parameter estimation. For example, while we may design experiments towards a particular value of each parameter, will oscillations disappear if our estimates of the parameters are slightly inaccurate? In order to address this question, we performed case studies of a random and arbitrarily chosen parameter set for both the bounded and unbounded self-assembly systems (Figs. S2 and S3, respectively). In particular, we used the first parameter set found using random sampling to yield oscillations. We then varied the parameters one by one, keeping all other parameters fixed, and measured the effects of these parameter variations on the presence and period of oscillations. Yellow points represent values of the parameter for which the system does not exhibit sustained oscillations; blue curves represent how the period of oscillation changes as a result of parameter variation.

For the bounded self-assembly system, we find that with the exception of the concentrations κ_{tot} and ρ_{tot}, oscillations are robust to even an order-of-magnitude error in parameter estimation. In addition, oscillations are robust to approximately 5-fold errors in κ_{tot} or ρ_{tot}. We also find that arbitrarily small values of $\tilde{P}$ can yield oscillations. Finally, as expected from the main text discussion, variations in $k_{u\kappa}$ most strongly affect the periods of resulting oscillations. We verify that are results are not dependent on the small values of $k_{u\kappa}$ and $k_{u\rho}$ in the randomly chosen parameter set, by performing the same analysis on a second random parameter set (Fig. S2b).

For the unbounded self-assembly system, we find that oscillations are less robust to parameter variation in two random and arbitrarily chosen parameter sets. One exception is the parameter $k_{d\kappa}$ which was found in the second parameter set to be variable by nearly two orders of magnitude in the oscillatory regime. Our results suggest oscillations may be more robust in the bounded self-assembly system than in the unbounded system.

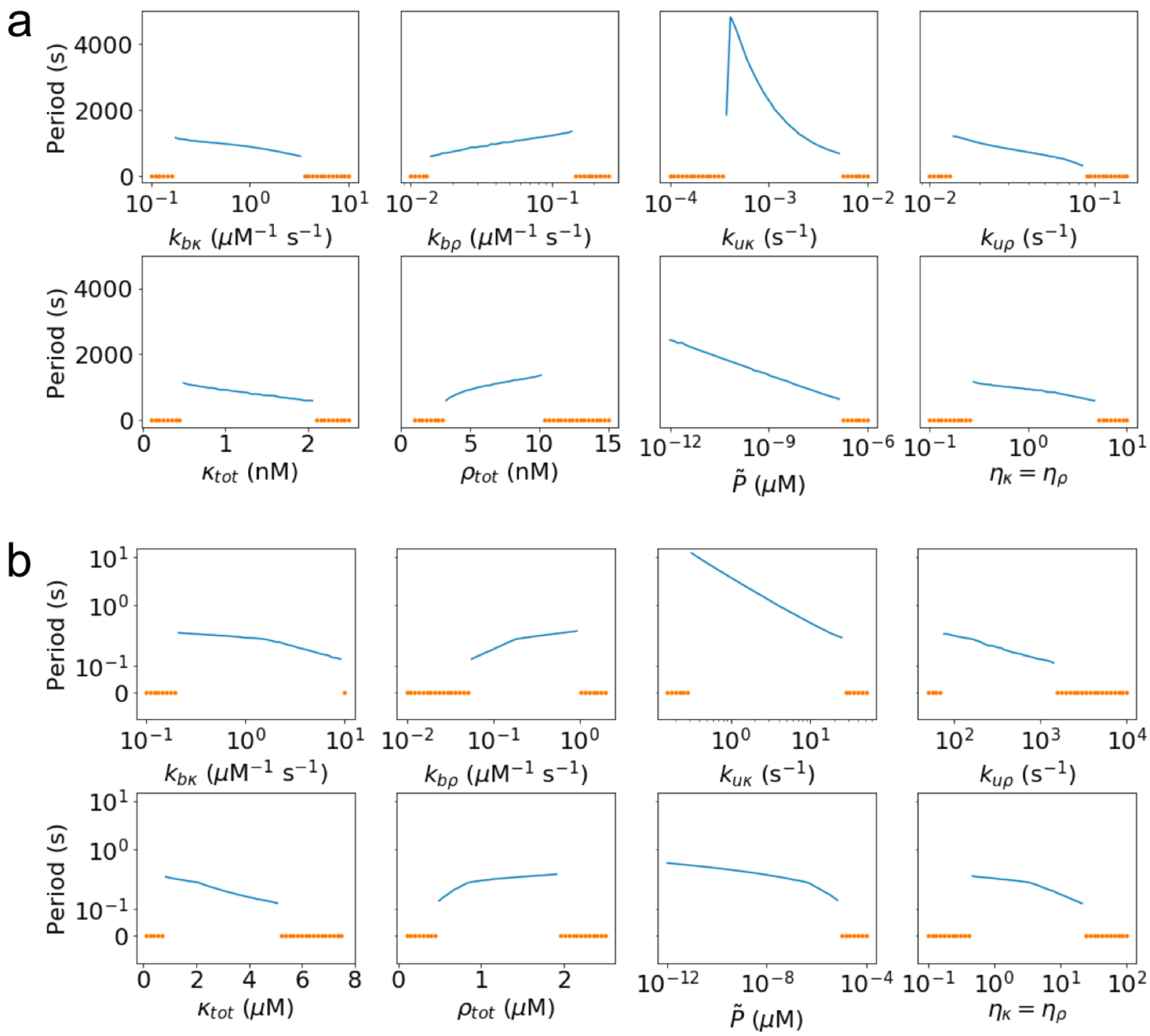

Figure S2: **Robustness analysis for bounded self-assembly system in the limit of fast enzymatic activity compared to self-assembly.** We consider the robustness of oscillations found for Eqn. 1. Yellow points represent values of the parameter for which the system does not exhibit sustained oscillations; blue curves represent how the period of oscillation changes as a result of parameter variation. Panel **a** uses the first oscillatory parameter set found. In order to verify that robustness of oscillations is not due to small values of $k_{u\kappa}$ and $k_{u\rho}$, panel **b** uses the first oscillatory parameter set found for which those values were both greater than 1 s^{-1}. In both parameter sets, oscillations are robust to over an order-of-magnitude variation in all parameters other than κ_{tot} and ρ_{tot}. All x-axes are in log-scale except for those two parameters. Arbitrarily small values of $\tilde{P}$ also give oscillations in both parameter sets. Finally, the period is heavily affected by $k_{u\kappa}$ as described in the main text.

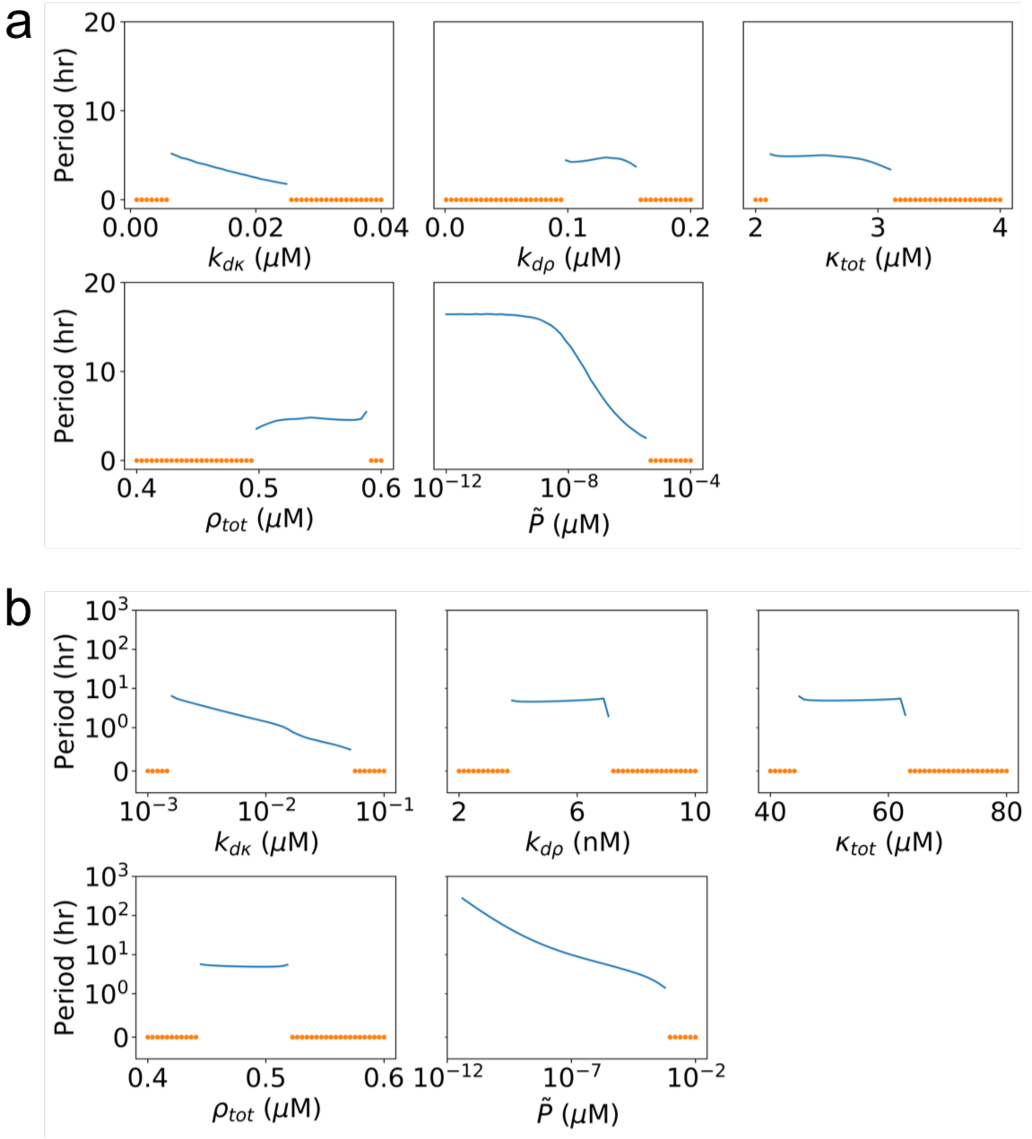

Figure S3: **Robustness analysis for unbounded self-assembly system.** We consider the robustness of oscillations found for Eqn. 9. Yellow points represent values of the parameter for which the system does not exhibit sustained oscillations; blue curve represents how the period of oscillation changes as a result of parameter variation. Panels **a** and **b** use the first two oscillatory parameter sets found. Oscillations appear less robust to parameter variations than in the bounded self-assembly system (Fig. S2), with the exception of the parameter $k_{d\kappa}$ in the second parameter set which can vary by nearly two orders of magnitude in the oscillatory regime. In addition, as in Fig. S2, arbitrarily small values of $\tilde{P}$ also give oscillations in both parameter sets.

S4 Considering the limit of fast self-assembly compared to enzymatic activity in the bounded self-assembly system

In the bounded self-assembly system, the limit corresponding to fast self-assembly compared to phosphorylation/dephosphorylation corresponds to the assumption

$$
\begin{aligned}
k_{b\kappa}\kappa^n &= k_{u\kappa}K \\
k_{b\rho}\rho^m &= k_{u\rho}P
\end{aligned}
\tag{S11}
$$

Using this assumption, we can describe the system dynamics using a set of two coupled differential equations. Defining $k_{d\kappa} = k_{u\kappa}/k_{b\kappa}$, and similarly for ρ

$$
\begin{aligned}
\frac{d\kappa}{dt} &= -\eta_{K\kappa}\frac{\kappa^{n+1}}{k_{d\kappa}} + \eta_{P\kappa}\left(\kappa_{\text{tot}} - \kappa - n\frac{\kappa^n}{k_{d\kappa}}\right)\left(\frac{\rho^m}{k_{d\rho}} + \tilde{P}\right) \\
\frac{d\rho}{dt} &= -\eta_{K\rho}\rho\frac{\kappa^n}{k_{d\kappa}} + \eta_{P\rho}\left(\rho_{\text{tot}} - \rho - m\frac{\rho^m}{k_{d\rho}}\right)\left(\frac{\rho^m}{k_{d\rho}} + \tilde{P}\right).
\end{aligned}
\tag{S12}
$$

Our goal is to determine whether this system can give rise to oscillations. Oscillations necessitate positive real parts of the Jacobian of the system evaluated at the fixed point, corresponding to positive values of its trace. The trace of the Jacobian is given by

$$
\begin{aligned}
\text{tr}(J) =& \frac{\partial\dot{\kappa}}{\partial\kappa} + \frac{\partial\dot{\rho}}{\partial\rho} \\
=& -\eta_{K\kappa}(n+1)\frac{\kappa^n}{k_{d\kappa}} - \eta_{P\kappa}\left(\frac{\rho^m}{k_{d\rho}} + \tilde{P}\right)\left(n^2\frac{\kappa^{n-1}}{k_{d\kappa}} + 1\right) \\
& - \eta_{K\rho}\frac{\kappa^n}{k_{d\kappa}} - \eta_{P\rho}\left((m+1)\frac{\rho^m}{k_{d\rho}} + \tilde{P} + \frac{2}{\rho}\left(\frac{m\rho^m}{k_{d\rho}}\right)^2 + m^2\frac{\rho^{m-1}}{k_{d\rho}}\tilde{P} - m\frac{\rho^{m-1}}{k_{d\rho}}\rho_{\text{tot}}\right).
\end{aligned}
\tag{S13}
$$

where here and in the rest of the section, all concentrations are measured at the fixed point of Eqn. S12.

All terms in the trace are negative except for the last. Therefore, the system has the potential to oscillate only if the final term is larger in magnitude than all the rest combined.

To simplify, we notice that given our experimental constraints that $\eta_{K\kappa} = \eta_{K\rho}$ and $\eta_{P\kappa} = \eta_{P\rho}$, the following holds at the fixed point of Eqn. S12:

$$\frac{\eta_{K\rho}}{\eta_{P\rho}} \frac{K}{P + \tilde{P}} = \frac{\kappa^p}{\kappa} = \frac{\rho^p}{\rho}. \tag{S14}$$

Using these equalities and substituting in Eqn. S11 as well as the conservation law $\rho_{\text{tot}} = \rho^p + \rho + mP$, we find after some algebra that

$$\text{tr}(J) = -\eta_{K\rho} K \left(-m \frac{P}{P + \tilde{P}} + 2 + n + n^2 \frac{K}{\kappa^p} + 2 \frac{\kappa}{\kappa^p} + m^2 \frac{P}{\rho^p} \right). \tag{S15}$$

In this form, it is clear that oscillations are not possible if $m < n + 3$. Oscillations are also not possible if the fixed point concentration of self-assembled phosphatase is not much larger than the concentration of constitutive phosphatase. However, this equation does not rule out oscillations for large values of m. We proceed by searching for oscillations numerically.

As in our other numerical studies in this work, we logarithmically sample random parameters. However, here, we also allow m to vary, as a randomly (uniformly) chosen integer between 5 and 20, while maintaining $n = 2$. We set the total concentrations of monomers to be between 10^{-4} and 100 μM and allow $\tilde{P}$ to vary between $10^{-6}/m$ μM and $\rho_{\text{tot}}/10m$. For this numerical study, we rescale time by a factor of $\eta_{K\rho} \times \mu$M. We choose values of k_d between 10^{-5} and 10^5 μM^{m-1}. We integrate each parameter set up to time 10^5 in rescaled time units; we found that 100 random parameter sets all reached steady state within a tenth of that time.

We examined 2×10^6 parameter sets randomly sampled in this fashion, and found 10 sets yielding oscillations for Eqn. S12. For comparison, we found 1717 out of 5×10^4 parameter sets yielding oscillations for Eqn. 1. The parameter sets yielding oscillations are given in Table S1. All oscillation periods found were far from 10^4, demonstrating that our integration time limit did not play a role in limiting oscillations found. The oscillating parameter sets found require extremely large values of m, beyond what is currently readily accessible experimentally. We

Index	n	m	η	$k_{d\kappa}$ (μM)	$k_{d\rho}$ (μM^{m-1})	κ_{tot} (μM)	ρ_{tot} (μM)	$\tilde{P}$ (μM)	Period
1	2	14	1.1	41.5	201	36.1	9.23	0.0591	5.82
2	2	12	1.1	68.8	0.0235	42.8	4.80	0.0309	17.0
3	2	17	1.1	979	11800	58.7	6.94	0.0322	12.8
4	2	15	1.1	399	0.00540	56.7	3.82	0.0193	40.3
5	2	9	1.1	124	5.15	96.3	9.11	0.0391	18.4
6	2	10	1.1	21.6	3.89e-5	50.5	3.75	0.0292	5.50
7	2	10	1.1	1090	2.60e-5	49.9	1.37	0.00710	56.8
8	2	20	1.1	26.4	0.434	56.5	10.6	0.0516	4.3
9	2	14	1.1	626	4.99e-4	32.7	2.20	0.0104	85.9
10	2	9	1.1	36.6	0.00308	85.6	5.92	0.0315	6.05

Table S1: Parameter sets found to yield oscillations in Eqn. S12, of 2×10^6 examined. The period is measured in the rescaled time units described in the main text.

analyze the robustness of the oscillations to one-dimensional variations in parameters in Fig. S4. Given the relative paucity of oscillating solutions found by random sampling and their apparent relative fragility (compared to Fig. S2) as well as the technical difficulties associated with implementing these solutions in the lab (in particular, with achieving robust homomultimeric self-assembly with large numbers of monomers per multimer), the limit of fast self-assembly compared to enzymatic activity appears less promising than the opposite limit considered in the main text of this work.

We do not consider here hybrid systems where the kinase self-assembly is bounded while the phosphatase is unbounded (or vice versa) though this analysis suggests that in the limit of fast self-assembly, such systems may be worth examining in greater detail.

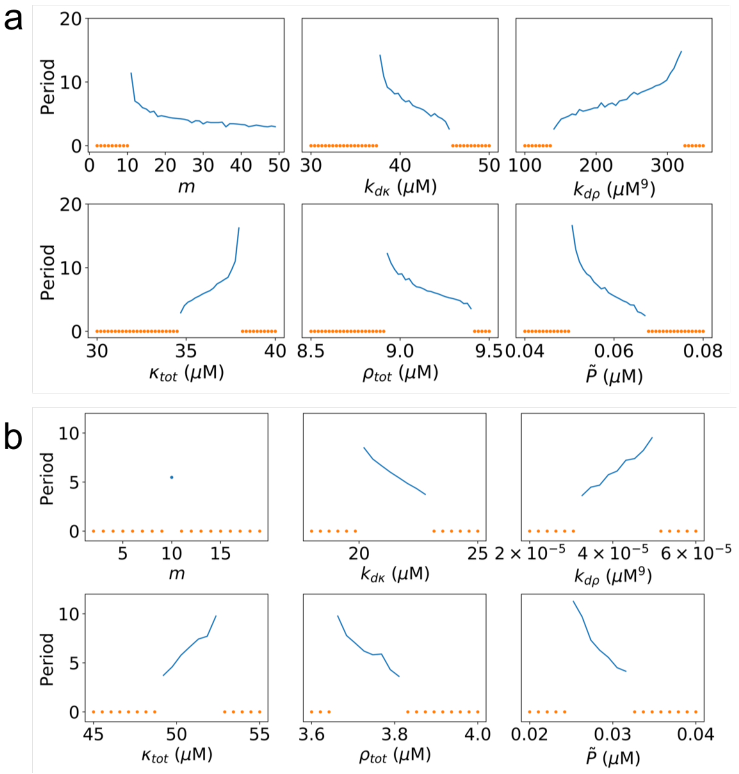

Figure S4: **Robustness analysis for bounded self-assembly system in the limit of fast self-assembly.** In panel **a** we perform robustness analysis as in Figs. S2 and S3 using the first parameter set found yielding oscillations (top row of Table S1). We find that after a threshold at $m = 11$, larger values of m do not appear to preclude oscillations, and oscillation period appears to plateau for large values of m. Oscillations appear most robust to variations in $k_{d\rho}$, which can vary by a factor of ~ 2 in the oscillatory regime. In contrast, all other parameters can vary only by a fraction of their value while maintaining oscillations. In panel **b** we verify similar features in the robustness plot of another randomly and arbitrarily chosen parameter set (the first oscillation found in the second 10^6 parameters screened; sixth row of Table S1); the one significant difference found was that in that parameter set, only $m = 10$ yielded oscillations. These results should be compared to Fig. S2 which show that in the separation-of-timescales limit explored in the main text, oscillations are far more robust: all non-concentration parameters can vary by over an order of magnitude in the oscillatory regime. The relative fragility of oscillations shown in this figure also helps explain the relative paucity of oscillations found using random sampling.

S5 Numerical search for oscillations

In order to determine if a parameter set leads to oscillations, we numerically integrated the differential equations. For bounded self-assembly, we used initial conditions of $(K, P) = (0, 0)$, and for unbounded, $(k, p) = (2\frac{\kappa_{\text{tot}}}{k_{d\kappa}}, 2\frac{\rho_{\text{tot}}}{k_{d\rho}})$. We integrated up to a time determined by the inverse of the minimum timescale in the system. For bounded self-assembly, we integrated up to a time $t_{\text{max}} = 10^3/\min\left(k_{u\kappa}, k_{u\rho}, k_{b\kappa}\kappa_{\text{tot}}^{n-1}, k_{b\rho}\rho_{\text{tot}}^{m-1}\right)$, while for unbounded, we used $t_{\text{max}} = 10^7/\min\left(\eta_{K\kappa}\kappa_{\text{tot}}, \eta_{K\rho}\kappa_{\text{tot}}, \eta_{P\kappa}\rho_{\text{tot}}, \eta_{P\rho}\rho_{\text{tot}}\right)$. For the full system of equations for bounded self-assembly, we used initial conditions corresponding to fully unphosphorylated and unbound κ, ρ, and $\tilde{P}$, and integrated up to

$$t_{\text{max}} = 10^4/\min\left(k_{u\kappa}, k_{u\rho}, k_{b\kappa}\kappa_{\text{tot}}^{n-1}, k_{b\rho}\rho_{\text{tot}}^{m-1}, k_{cK\kappa}, k_{cP\kappa}, k_{cK\rho}, k_{cP\rho}, k_{bK\kappa}\kappa_{\text{tot}}, k_{bP\kappa}\tilde{P}_{\text{tot}}\right).$$

We set the enzyme dissociation constants $k_{uK\kappa}$, $k_{uP\kappa}$, $k_{uK\rho}$, $k_{uP\kappa}$ equal to their respective catalytic rate constants, since the former are largely unspecified by constraints on binding rates and Michaelis constants. Our results are largely insensitive to this assumption. In all cases, the prefactors for t_{max} were determined by applying an order of magnitude larger prefactor and finding no new oscillating solutions.

To determine if the results of the numerical integration can be labeled as oscillations, we used a set of heuristics. We verified these heuristics by plotting solutions found by them to produce oscillations and finding no evidence of false positives. These heuristics considered the behavior of a single system component (e.g. K for bounded self-assembly). First, we determined whether the number of inflection points in the solution is greater than 10. Second, to weed out decaying oscillations, whether the smallest amount by which the component changed between inflection points and the amount it changed between an arbitrarily chosen set of inflection points (between the fifth and sixth) is within $2\times$. Also to weed out decaying oscillations, we measured the amount the component changed between a set of inflection points around the

$3t_{\text{max}}/4$ mark–let's call this amount $x_{3/4}$–and between the penultimate and final inflection point, x_1. We verified that $|(x_{3/4} - x_1)/x_1| < 1$, meaning that the relative change in oscillating height was no more than 100%. We also considered whether the solver required sampling points at a significant frequency (to weed out numerical oscillations): we used the criterion that the third-to-last sampled time point was within 5% of the second-to-last sampled time point. To further root out spurious numerical oscillations we measured the period of the oscillation in two ways–as the time between the third-to-last and last inflection point, and as between the fifth-to-last and third-to-last–and verified that they differed by no more than 1% (parameter sets that fail the equal-period test were integrated for $10\times$ longer and re-tested). Finally, we examined the numerical solution by eye for all parameter sets found to produce oscillations, in order to verify that even if our heuristics produce false negatives (of which we have found almost no evidence) our results contain no false positives.

S6 Supplementary figures

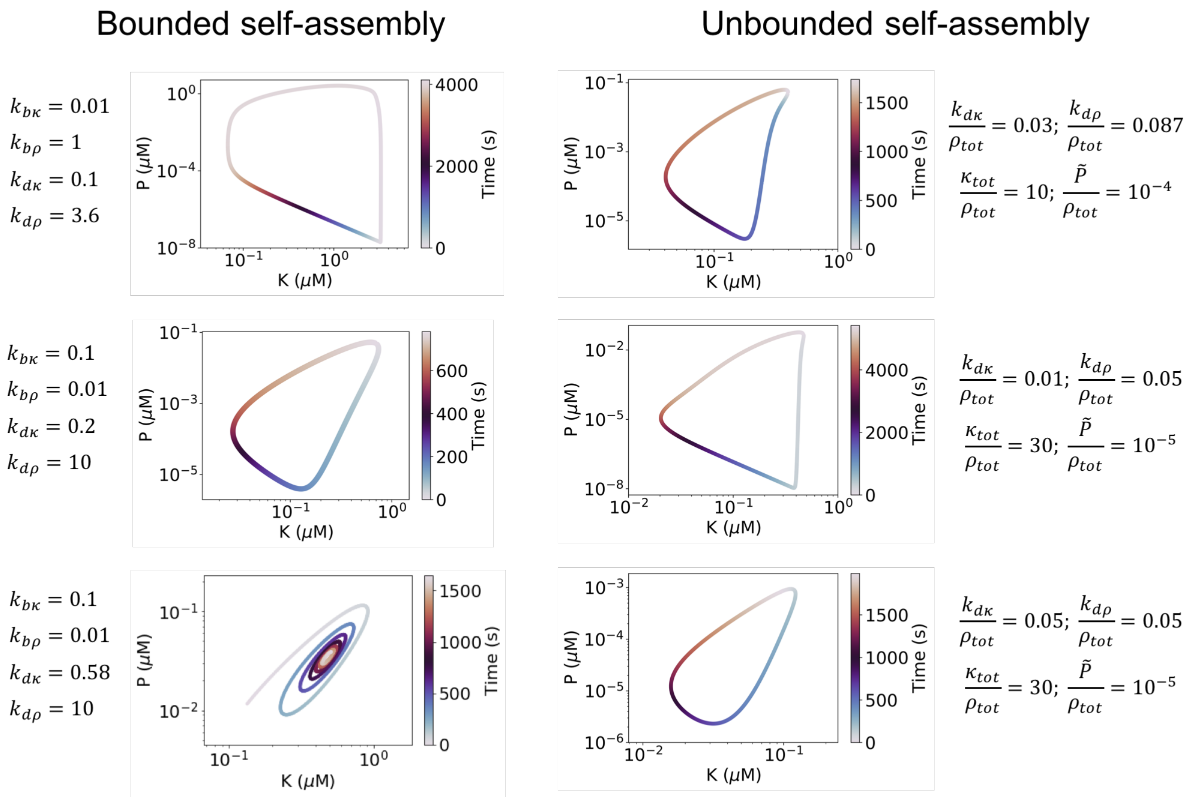

Figure S5: **Example trajectories**. Trajectories displayed in Figs. 2 and 4b are shown along with the parameters used for each trajectory. For trajectories showing sustained oscillations (all but the lower left, which shows a decaying oscillation, and therefore not a desired trajectory) one oscillation cycle is shown.

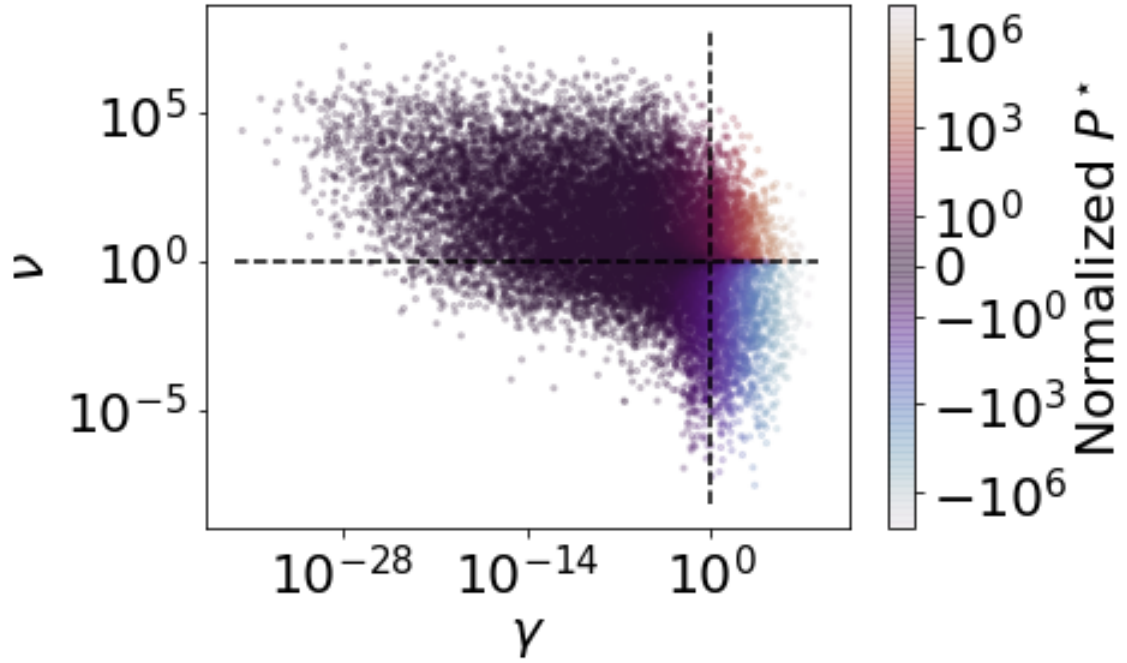

Figure S6: **Verifying Eqn. 4**. Here we plot the parameter sets shown in Fig. 3b in the same phase diagram as that figure, but colorcoded by the normalized steady-state concentration of phosphatase multimer, found using Python's scipy.optimize.root function. In particular, color-coding is given by the left-hand-side of Eqn. 4. Thus, for each parameter set, we show the fixed point concentration of the phosphatase multimer, normalized by the constitutive phosphatase concentration and by a function of the unbinding rates of kinase and phosphatase multimers. The results of this figure demonstrate the validity of our analytical analysis. For large values of γ, the absolute value of normalized $P^\star$ is large; meanwhile, the sign of the normalized $P^\star$ is positive for $\nu > 1$ and negative otherwise. Since oscillations require the normalized value of $P^\star$ to be greater than unity, oscillations are restricted to the upper right quadrant of the phase diagram.

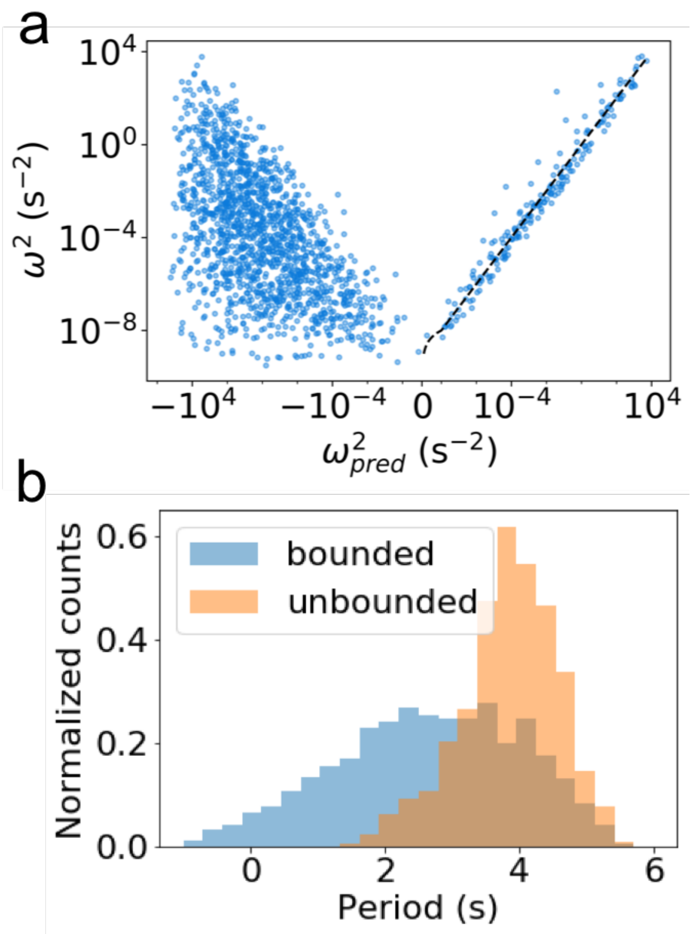

Figure S7: **Oscillation periods.** **a:** Numerical integration of Eqn. 1 demonstrates Eqn. 6 correctly predicts the frequency of oscillations for the bounded self-assembly system in the linear regime around the fixed point, but is not predictive outside this regime. We make no constraints on the fixed points of the parameter sets considered here. The x-axis shows the predicted squared frequency while the y-axis shows the true squared frequency. For $\omega^2_{\text{pred}} > 0$, the two formulae agree (black dashed line represents $\omega^2 = \omega^2_{\text{pred}}$). For $\omega^2_{\text{pred}} < 0$, ω^2_{pred} is no longer predictive since the oscillations cannot be understood through linear stability analysis of the fixed point. **b:** Random parameters logarithmically distributed within the experimental regime were sampled for Eqns. 1 (bounded self-assembly; blue) and 9 (unbounded self-assembly; orange). The periods of resulting oscillations are histogrammed logarithmically, showing a possible range of periods spanning orders of magnitude, from fractions of a second (minute) for bounded (unbounded) self-assembly, to > 1 day.

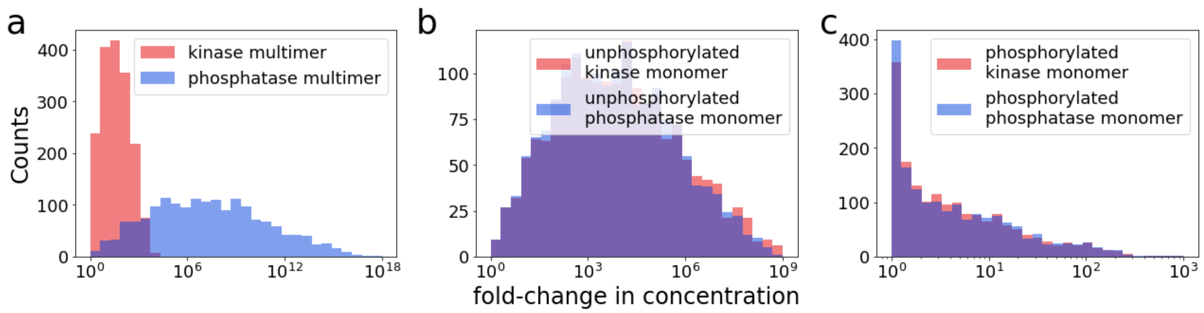

Figure S8: **Amplitude analysis for bounded self-assembly system.** Various experimental tools can be used to visualize the system oscillations. As just two examples, a fluorophore-quencher pair on complementary monomers can enable the visualization of oscillations in monomer concentration, while split fluorophores can enable the visualization of multimer concentrations. Here we numerically integrate oscillating parameter sets found for Eqn. 1, and histogram the amplitude of oscillations of various system components. We define the amplitude here as the maximum concentration divided by the minimum concentration across an oscillatory cycle. We perform this analysis for kinase and phosphatase multimers, unphosphorylated monomers, and phosphorylated monomers. Our results show that typical oscillations involve variation of many orders of magnitude in the concentrations of phosphatase multimers, as well as unphosphorylated kinase and phosphatase monomers. However, these results suggest oscillations cannot be readily visualized using only the concentrations of phosphorylated monomers.

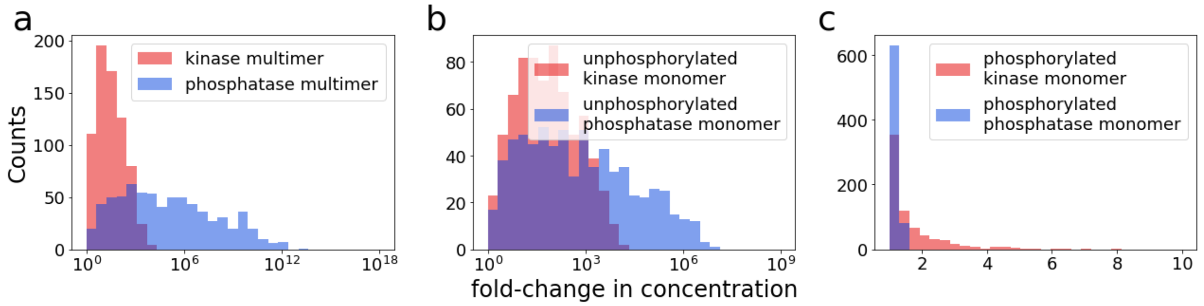

Figure S9: **Amplitude analysis for unbounded self-assembly system.** Analysis performed as in Fig. S8. Our results suggest that oscillations in the concentrations of phosphatase multimers and unphosphorylated monomers should be most readily visible, as they typically vary by several orders of magnitude over an oscillation cycle. Concentrations of kinase multimers and unphosphorylated monomers also typically vary by at least an order of magnitude over an oscillation cycle. Concentrations of phosphorylated monomers typically do not vary by much.

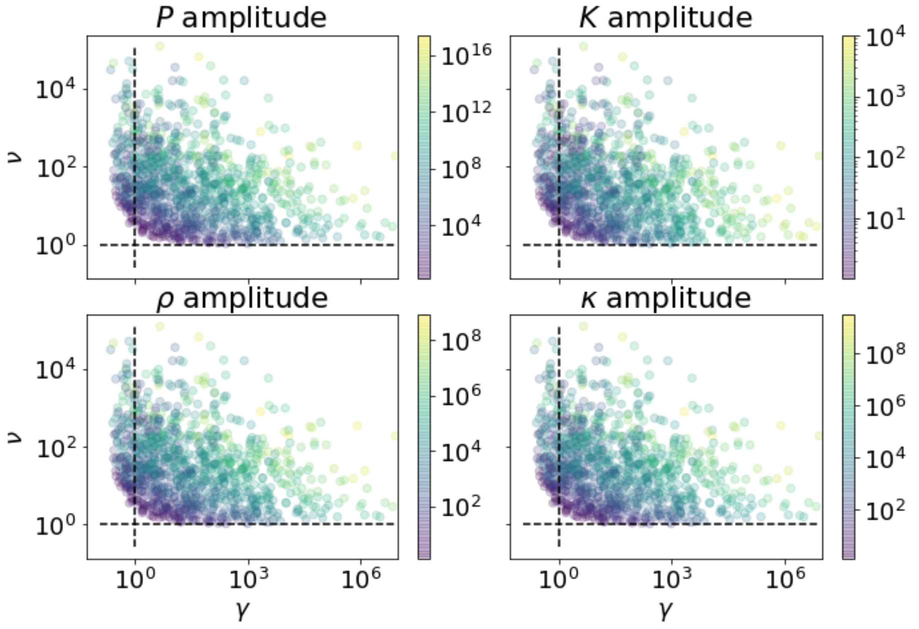

Figure S10: **Amplitudes of bounded self-assembly system oscillations are larger farther from the bifurcation threshold.** Analysis performed as in Fig. S8. Here we show the oscillating parameter sets in γ-ν space, colorcoded by the fold-change in concentration of P, K, ρ, or κ over an oscillatory cycle. Our results demonstrate that parameter sets farther from either bifurcation point ($\gamma = 1$ or $\nu = 1$) typically result in higher amplitudes of oscillations.

REFERENCES AND NOTES

1. O. Purcell, N. J. Savery, C. S. Grierson, M. di Bernardo, A comparative analysis of synthetic genetic oscillators. *J. R. Soc. Int.* **7**, 1503–1524 (2010).

2. M. W. Young, S. A. Kay, Time zones: A comparative genetics of circadian clocks. *Nat. Rev. Genet.* **2**, 702–715 (2001).

3. D. Bray, Protein molecules as computational elements in living cells. *Nature* **376**, 307–312 (1995).

4. X. J. Gao, L. S. Chong, M. S. Kim, M. B. Elowitz, Programmable protein circuits in living cells. *Science* **1258**, 1252–1258 (2018).

5. T. Fink, J. Lonzarić, A. Praznik, T. Plaper, E. Merljak, K. Leben, N. Jerala, T. Lebar, Ž. Strmšek, F. Lapenta, M. Benčina, R. Jerala, Design of fast proteolysis-based signaling and logic circuits in mammalian cells. *Nat. Chem. Biol.* **15**, 115–122 (2018).

6. B. Novák, J. J. Tyson, Design principles of biochemical oscillators. *Nat. Rev. Mol. Cell Biol.* **9**, 981–991 (2008).

7. C. C. Jolley, K. L. Ode, H. R. Ueda, A design principle for a posttranslational biochemical oscillator. *Cell Rep.* **2**, 938–950 (2012).

8. M. Nakajima, K. Imai, H. Ito, T. Nishiwaki, Y. Murayama, H. Iwasaki, T. Oyama, T. Kondo, Reconstitution of circadian oscillation of cyanobacterial KaiC phosphorylation in vitro. *Science* **308**, 414–415 (2005).

9. J. Michael, J. S. Markson, W. S. Lane, D. S. Fisher, E. K. O'Shea, Ordered phosphorylation governs oscillation of a three-protein circadian clock. *Science* **318**, 809–812 (2007).

10. M. J. Rust, Orderly wheels of the cyanobacterial clock. *Proc. Natl. Acad. Sci. U.S.A.* **109**, 16760–16761 (2012).

11. H. Kageyama, T. Nishiwaki, M. Nakajima, H. Iwasaki, T. Oyama, T. Kondo, Cyanobacterial circadian pacemaker: Kai protein complex dynamics in the KaiC phosphorylation cycle in vitro. *Mol. Cell* **23**, 161–171 (2006).

12. S. E. Boyken, M. A. Benhaim, F. Busch, M. Jia, M. J. Bick, H. Choi, J. C. Klima, Z. Chen, C. Walkey, A. Mileant, A. Sahasrabuddhe, K. Y. Wei, E. A. Hodge, S. Byron, A. Quijano-Rubio, B. Sankaran, N. P. King, J. Lippincott-Schwartz, V. H. Wysocki, K. K. Lee, D. Baker, De novo design of tunable, pH-driven conformational changes. *Science* **364**, 658–664 (2019).

13. B. Kholodenko, Negative feedback and ultrasensitivity can bring about oscillations in the mitogen-activated protein kinase cascades. *Eur. J. Biochem.* **267**, 1583–1588 (2000).

14. M. Strumillo, P. Beltrao, Towards the computational design of protein posttranslational regulation. *Bioorg. Med. Chem.* **23**, 2877–2882 (2015).

15. L. Doyle, J. Hallinan, J. Bolduc, F. Parmeggiani, D. Baker, B. L. Stoddard, P. Bradley, Rational design of α-helical tandem repeat proteins with closed architectures. *Nature* **528**, 585–588 (2015).

16. H. Nishi, K. Hashimoto, A. R. Panchenko, Phosphorylation in protein-protein binding: Effect on stability and function. *Structure* **19**, 1807–1815 (2011).

17. H. Nishi, A. Shaytan, A. R. Panchenko, Physicochemical mechanisms of protein regulation by phosphorylation. *Front. Genet.* **5**, 270 (2014).

18. K. Camacho-Soto, J. Castillo-Montoya, B. Tye, I. Ghosh, Ligand-gated split-kinases. *J. Am. Chem. Soc.* **136**, 3995–4002 (2014).

19. K. Camacho-Soto, J. Castillo-Montoya, B. Tye, L. O. Ogunleye, I. Ghosh, Small molecule gated split-tyrosine phosphatases and orthogonal split-tyrosine kinases. *J. Am. Chem. Soc.* **136**, 17078–17086 (2014).

20. Z. Li, S. Liu, Q. Yang, Incoherent inputs enhance the robustness of biological oscillators. *Cell Syst.* **5**, 72–81.e4 (2017).

21. P. Smolen, Frequency selectivity, multistability, and oscillations emerge from models of genetic regulatory systems. *Am. J. Physiol.* **274**, C531–C542 (1998).

22. J. Hasty, M. Dolnik, V. Rottschäfer, J. J. Collins, Synthetic gene network for entraining and amplifying cellular oscillations. *Phys. Rev. Lett.* **88**, 148101 (2002).

23. J. Stricker, S. Cookson, M. R. Bennett, W. H. Mather, L. S. Tsimring, J. Hasty, A fast, robust and tunable synthetic gene oscillator. *Nature* **456**, 516–519 (2008).

24. M. Schlosshauer, D. Baker, Realistic protein-protein association rates from a simple diffusional model neglecting long-range interactions, free energy barriers, and landscape ruggedness. *Protein Sci.* **13**, 1660–1669 (2004).

25. S. E. A. Ozbabacan, H. B. Engin, A. Gursoy, O. Keskin, Transient protein–protein interactions. *Protein Eng. Des. Sel.* **24**, 635–648 (2011).

26. S. Zhuo, J. C. Clemens, R. L. Stone, J. E. Dixon, Mutational analysis of a Ser/Thr phosphatase. *J. Biol. Chem.* **269**, 26234–26238 (1994).

27. C. Chen, B. H. Ha, A. F. Thévenin, H. J. Lou, R. Zhang, K. Y. Yip, J. R. Peterson, M. Gerstein, P. M. Kim, P. Filippakopoulos, S. Knapp, T. J. Boggon, B. E. Turk, Identification of a major determinant for serine-threonine kinase phosphoacceptor specificity. *Mol. Cell* **53**, 140–147 (2014).

28. R. A. Langan, S. E. Boyken, A. H. Ng, J. A. Samson, G. Dods, A. M. Westbrook, T. H. Nguyen, M. J. Lajoie, Z. Chen, S. Berger, V. K. Mulligan, J. E. Dueber, W. R. P. Novak, H. El-Samad, D. Baker, De novo design of bioactive protein switches. *Nature* **572**, 205–210 (2019).

29. A. H. Ng, T. H. Nguyen, M. Gómez-Schiavon, G. Dods, R. A. Langan, S. E. Boyken, J. A. Samson, L. M. Waldburger, J. E. Dueber, D. Baker, H. El-Samad, Modular and tunable biological feedback control using a de novo protein switch. *Nature* **572**, 265–269 (2019).
